# Discovery of serum biomarkers of ovarian cancer using complementary proteomic profiling strategies

**DOI:** 10.1002/prca.201400063

**Published:** 2014-11-10

**Authors:** John F. Timms, Elif Arslan‐Low, Musarat Kabir, Jenny Worthington, Stephane Camuzeaux, John Sinclair, Joanna Szaub, Babak Afrough, Vladimir N. Podust, Evangelia‐Ourania Fourkala, Myriam Cubizolles, Florian Kronenberg, Eric T. Fung, Aleksandra Gentry‐Maharaj, Usha Menon, Ian Jacobs

**Affiliations:** ^1^ EGA Institute for Women's HealthUniversity College LondonLondonUK; ^2^ Vermillion Inc. (formerly Ciphergen Biosystems)AustinTexasUSA; ^3^ Division of Genetic EpidemiologyInnsbruck Medical UniversityInnsbruckAustria

**Keywords:** Diagnostic biomarkers, MS, Ovarian cancer, Serum profiling

## Abstract

**Purpose:**

Ovarian cancer is a devastating disease and biomarkers for its early diagnosis are urgently required. Serum may be a valuable source of biomarkers that may be revealed by proteomic profiling. Herein, complementary serum protein profiling strategies were employed for discovery of biomarkers that could discriminate cases of malignant and benign ovarian cancer.

**Experimental design:**

Identically collected and processed serum samples from 22 cases of invasive epithelial ovarian cancer, 45 benign ovarian neoplasms, and 64 healthy volunteers were subjected to immunodepletion and protein equalization coupled to 2D‐DIGE/MS and multidimensional fractionation coupled to SELDI‐TOF profiling with MS/MS for protein identification. Selected candidates were verified by ELISA in samples from malignant (*n* = 70) and benign (*n* = 89) cases and combined marker panels tested against serum CA125.

**Results:**

Both profiling platforms were complementary in identifying biomarker candidates, four of which (A1AT, SLPI, APOA4, VDBP) significantly discriminated malignant from benign cases. However, no combination of markers was as good as CA125 for diagnostic accuracy. SLPI was further tested as an early marker using prediagnosis serum samples. While it rose in cases toward diagnosis, it did not discriminate prediagnosis cases from controls.

**Conclusions and clinical relevance:**

The candidate biomarkers warrant further validation in independent sample sets.

**Abbreviations**
ROC: receiver operating characteristic
UKCTOCS: UK Collaborative Trial of Ovarian Cancer Screening
UKOPS: United Kingdom Ovarian Cancer Population Study
VDBP: vitamin D binding protein

## 1 Introduction

Every year there are over 200 000 new cases of ovarian cancer and 125 000 deaths worldwide 1. The prognosis is poor reflecting the late stage at diagnosis and lack of an established screening programme. Transvaginal ultrasound and serum CA125 are the main diagnostic tests for primary invasive epithelial ovarian/tubal cancers in symptomatic patients. However, CA125 is not cancer‐specific and may be elevated in benign gynecological conditions such as benign ovarian neoplasms and endometriosis, necessitating further investigation by imaging 2. In addition, not all early‐stage tumors generate sufficient CA125 for diagnostic purposes. Consequently, biomarkers with improved accuracy for detecting and diagnosing ovarian cancer are urgently required. While several marker panels and algorithms have been reported for differential diagnosis 3, 4, 5, 6, 7, 8, 9, 10, 11, there is as yet insufficient evidence to support their wider clinical use, particularly for

**Clinical Relevance**

We have used two complementary serum protein profiling strategies to identify novel biomarkers for the differential diagnosis of ovarian cancer and further tested one of these as an early detection marker using prediagnosis samples. Markers SLPI, VDBP, A1AT, and APOA4 all significantly discriminated malignant from benign cases, although did not add to CA125 in multimarker models in this cohort of patients where CA125 performed exceptionally well. One of the candidate markers, SLPI, was raised in the lead up to diagnosis using prediagnostic samples, but did not significantly discriminate cases from matched controls. The further validation of these candidate biomarkers in more representative patient cohorts is warranted.

early detection of ovarian cancer. It is noteworthy that several of these panels include CA125, which provides much of the discriminating power. Notably, the serum marker HE4 holds promise and appears to complement CA125 in discriminating cancer from benign conditions in premenopausal women.

Analysis of serum/plasma proteomes for biomarker discovery remains challenging owing to the extreme dynamic range of expression of the constituent proteins and the fact that a small number of highly abundant serum proteins make up the majority of protein content. This has prompted researchers to use multidimensional protein/peptide separations and depletion methods as part of profiling strategies. Despite this, proteomic coverage is still limited and few if any robust cancer biomarkers have been identified using classical proteomic profiling methods. The need for identical handling of clinical samples is also of paramount importance in order to avoid largely proteolysis‐driven changes unrelated to biological variation and has been highlighted by numerous studies 12, 13, 14, 15, 16, 17. Finally, issues related to reproducibility and the robustness of class‐discriminating algorithms used for proteomic biomarker discovery have also been raised 18, 19.

Of the technologies employed, MALDI‐TOF MS and SELDI‐TOF MS have the capability for high‐throughput profiling when linked to (semi‐)automated sample processing, though lack resolution and mass accuracy, as well as dynamic range and sensitivity, particularly when analyzing complex samples such as body fluids. LC‐MS/MS provides higher mass accuracy and resolution, though it is inherently low throughput. Similarly, quantitative 2DE, for example 2D‐DIGE, is a low‐throughput technique, but has high quantitative accuracy, multiplexing capacity, a broad linear dynamic range of detection, and permits visualization of protein isoforms 20.

Herein, we have used two unbiased, complementary proteomic approaches to profile a case control set of serum samples from the UK Ovarian Cancer Population Study (UKOPS), collected and handled according to a rigorous SOP. Immunodepletion of the seven most abundant proteins and protein equalization technology was applied to improve coverage by 2D‐DIGE profiling, while a semiautomated multidimensional protein fractionation strategy incorporating protein equalization was linked to SELDI‐TOF profiling. Data were filtered to find potential biomarkers that could discriminate cases of malignant epithelial ovarian cancer from benign or healthy controls. Several of the candidates were further validated using ELISA and compared with CA125.

## 2 Materials and methods

### 2.1 Subjects, sample collection, and handling

This biomarker discovery study was approved by the Joint UCL/UCLH Committees on the Ethics of Human Research (Committee A) (Reference No. 05/Q0505/58). Written informed consent was obtained from all donors and no data allowing identification of patients was provided. Women were recruited into the UK Ovarian Cancer Population Study (UKOPS) from ten NHS Trusts across the UK through gynaecological oncology departments. Serum samples from patients were collected between 2005 and 2009 prior to surgery for an ovarian neoplasm, and the diagnosis of malignant or benign ovarian neoplasm was confirmed by independent review of notes and histopathology reports. Serum samples came from women diagnosed with primary invasive epithelial ovarian cancer (*n* = 70) or benign ovarian neoplasms (*n* = 89) and from healthy age‐matched controls (*n* = 173). Healthy volunteers were recruited from women attending annual screening in the UK Collaborative Trial of Ovarian Cancer Screening (UKCTOCS) 21, 22 also between 2005 and 2009. They had no family history of ovarian cancer and no diagnosis of a cancer during follow‐up. Median and mean ages, histological subtypes, and sample numbers for each group are detailed in Table 1. All samples were collected and processed identically as described 23.

**Table 1:** Case control samples used for study

	Malignant						Benign	Healthy
Histological subtype/patient group	Stage I (median age = 62.6; IQR 50.4–73.3)	Stage II (median age = 65.2; IQR 54.7–66.5)	Stage III (median age = 63.9; IQR 54.1–70.9)	Stage IV (median age = 69.2; IQR 59.3–73.1)	Unstaged (median age = 41.2)	Total (median age = 63.9; IQR 53.6–71.2)	Benign (median age = 58.7; IQR 48.7–68.5)	Healthy (median age = 63.9; IQR 59.2–69.1)
Serous carcinoma	5	2	23	4		34		
Endometrioid carcinoma	7	2	4		1	14		
Mucinous carcinoma	5		2			7		
Clear cell carcinoma	3	2	1	1		7		
Mixed carcinoma	3		1			4		
Transitional cell carcinoma	1					1		
Adenocarcinoma (unspecified)			3			2		
Borderline	9				2			
Benign							89	
Healthy								173
Total	33	6	34	5	3	70	89	173

FIGO staging was used. IQR, inter‐quartile range.

One candidate biomarker was also tested as an early marker in UKCTOCS prediagnosis samples and matched controls. Use of these samples was approved by the Joint UCL/UCLH Research Ethics Committee A (Ref. 05/Q0505/57), with written informed consent obtained from all donors and no data provided that allowed patient identification. Trial participants at enrolment were postmenopausal women aged 50–74 who had no family history of ovarian cancer. All participants were "flagged" with the national agencies for cancer registrations and deaths using their NHS number. Women subsequently diagnosed with ovarian cancer (cases) were identified by cross‐referencing with the Health and Social Care Information Centre cancer registry codes and death codes (ICD10 C56, C57.0) with diagnosis confirmed by review of histopathology reports. The study set comprised serum from women in the multimodal screening arm of UKCTOCS 22. There were two samples from each of 49 women taken 3–14 months ("late") and >32 months ("early") prior to diagnosis of primary invasive or borderline epithelial ovarian cancer that were subsequently grouped as Type I + Borderline (BL) and Type II, based on morphology and grade 24. Matched noncancer controls from 25 women (two samples each) were selected based on collection date, collection centre, and age for Type II cases. The resulting study set was 148 serum samples from 74 women, 19 of whom were subsequently diagnosed with Type I or BL ovarian cancer and 30 of whom were subsequently diagnosed with Type II ovarian cancer. Supporting Information Table 1 shows clinical, lifestyle, and sample data for this study set.

### 2.2 Serum profiling

Two complementary profiling strategies were used (detailed in Supporting Information Methods). The first used 2D‐DIGE analysis of a subset of 131 samples pooled (by equal volume) according to clinical group: healthy (*n* = 64), benign (*n* = 45), and malignant (*n* = 22). Three types of sample preparation were analyzed: unfractionated sera (UnF), sera immunodepleted of the seven most abundant proteins using the HPLC‐based Multiple Affinity Removal System (MARS; Agilent), and sera subjected to ProteoMiner (PM) Protein Enrichment (BioRad). The latter method relies on a large, highly diverse bead‐based library of combinatorial peptide ligands, which simultaneously dilutes high‐abundance proteins and concentrates low‐abundance proteins (protein equalization) to improve coverage.

The three sample preparations were labelled with Cy‐dyes and run in a series of 2D‐DIGE experiments to compare the proteomes of the healthy, benign, and malignant serum pools, essentially as described 25. Samples (50 or 100 μg protein) were run in quadruplicate with Cy3 and Cy5 dye swapping against a Cy2‐labeled standard run on each large‐format gel and comprised of a pool of all clinical groups for the relevant preparation method. Gel images were analyzed using DeCyder V5.0 software (GE Healthcare). Briefly, standardized volumes for spots‐matched across all gels were averaged across replicates for each condition and the ratios compared. Spots matching on all gels displaying a ≥1.5 average‐fold change in abundance with *p* values <0.05 (Student *t*‐test) were selected for automated picking, tryptic digestion, and extraction, as described 25.

The second profiling method used a proprietary, automated, multidimensional fractionation strategy (Deep Proteome; Ciphergen Biosystems) run on ProteinChip Arrays and coupled to high‐throughput SELDI‐TOF MS profiling. The method was applied to the 131 individual samples (64 healthy, 45 benign, and 22 malignant) run in triplicate and randomized across plates. Fractionation steps were performed in 96‐well filter plates on a vacuum manifold with liquid handling using a Tecan Aquarius robot with tip‐loader. A control serum sample (Intergen) was also run (*n* = 5) to monitor reproducibility. Briefly, 200 μL serum was denatured and incubated with Protein Equalizer Beads™ in 96‐well filter plates (Nunc). Flow‐through was collected followed by three elutions with high salt (E1), high organic (E2), and denaturing buffers (E3), respectively. The flow‐through was further fractionated by hydrophobic charge induction chromatography (using MEP HyperCel resin) into four fractions with flow‐through (FT) and guanidine‐HCl fractions (M3) retained for analysis. High organic/acid and urea/acid fractions were pooled and further separated by strong cation exchange chromatography (using ceramic S HyperD F resin) into four fractions (S1–S4). SDS‐denatured, unfractionated serum was also prepared for analysis (SDS). Thus, ten fractions were generated per sample in triplicate and applied to either or both of two SELDI chip types: CM10 (weak cation‐exchange) and/or IMAC30‐Cu2+ (immobilized metal affinity capture), depending on fraction type.

ProteinChip Arrays were read on a calibrated PCS4000 instrument (Ciphergen Biosystems) with automated chip loading, sample tracking, reading, and spectral processing controlled by Ciphergen Express software. Processing included spectral baseline definition, smoothing, and normalization based on total ion current. Calibrations were made using all‐in‐one protein and peptide calibrants at appropriate focus masses. Different focus masses were used for the samples and readings taken before and/or after formic acid treatment depending on SELDI chip and fraction type. This generated 30 profiles per sample in triplicate, resulting in a total of 11 790 spectra. Details of the method can be found in Supplementary Methods.

Spectra were processed and analyzed using the Expression Difference Mapping tool to define peaks (based on S/N, valley depth, and minimum peak thresholds), calculate peak intensities and align peaks across spectra. Univariate analysis of average peak intensities between clinical groups was performed using a Bonferroni‐corrected *p* value cut‐off (*p* < 0.000041) and an area under the ROC curve (AUC) cut‐off (<0.2 or >0.8) to define discriminating peaks. A cross‐bioprocessor/chip array comparison (irrespective of sample group) was also carried out to filter out false positives. Peaks were prioritized depending upon their ability to differentiate early‐stage or late‐stage cancers from benign and healthy controls. Multivariate analysis using classification trees was also performed on the filtered peak list to generate diagnostic models and correlation analysis used to identify commonly changing peaks.

### 2.3 MS‐based protein identification

Proteins from 2D‐DIGE gel spots were identified using MALDI‐TOF peptide mass fingerprinting (UnF and MARS1) or LC‐MS/MS peptide sequencing (MARS2 and PM) essentially as described 25, using the same stringent criteria for accepting positive identifications. In cases where multiple identifications were made from the same gel spots, all protein groups are reported. Identification of differential SELDI‐TOF peaks was carried out essentially as described 17. Briefly, proteins were fractionated by LC and ultrafiltration and identified either by direct MS/MS sequencing (<5 kDa) or purified by SDS‐PAGE prior to staining, excision, tryptic digestion, and MS/MS (>5 kDa). One quarter of each band was extracted from gels without digestion and reanalyzed by SELDI‐TOF MS to confirm matching of the band with the peak of interest. Samples were analyzed on a Q‐STAR XL equipped with a PCI‐1000 ProteinChip Interface (Ciphergen Biosystems) and data searched against the SwissProt or NCBInr databases. Peak identifications were also confirmed using data from previous publications (Supporting Information Table 3.3) and informed by correlation analysis using Spearman's rank test (Supporting Information Table 3.4).

### 2.4 ELISA validation

Serum CA125 concentrations were measured as part of UKOPS and UKCTOCS using a robust electro‐chemiluminescence sandwich immunoassay on an Elecsys 2010 analyzer (Roche Diagnostics) with the two monoclonal antibodies OC125 and M11 (Fujirebio Diagnostics). Apolipoprotein A‐IV (APOA4) was measured in duplicate using an in‐house developed double antibody ELISA as described 26, and using a 1:7500 dilution of serum. Other analytes were measured in serum in duplicate using commercial ELISA kits according to the manufacturers’ instructions. These were human vitamin D binding protein (VDBP) ELISA Kit (1:40 000 dilution; Immundiagnostik), human α1‐Antitrypsin (A1AT) ELISA Kit (1:240 000 dilution; Immundiagnostik), and Quantikine human secretory leukocyte protease inhibitor (SLPI) Immunoassay (1:20 dilution; R&D). For duplicate readings of the same samples, assays gave average CVs of 4.1% (CA125), 2.7% (APOA4), 13.8% (VDBP), 10.5% (A1AT), and 8.2% (SLPI). GraphPad Prism and Stata software were used for analysis of ELISA data and for combining markers by multivariate logistic regression. For normally distributed data, the Student *t* test was used to assess significance of differences; otherwise the Mann–Whitney *U*‐test was used. Fisher's exact test was used to assess significance of associations for noncontinuous variables and correlation analysis used Spearman's rank test. *p* values <0.05 were considered significant. Receiver operating characteristic (ROC) curves were constructed for each marker and their combinations from multivariate logistic regression analysis to assess diagnostic accuracy in differentiating malignant from benign neoplasms.

## 3 Results

### 3.1 2D‐DIGE/MS profiling for identification of candidate markers

A total of 934 matched protein features were detected in 2D‐DIGE images for the unfractionated sera (see Supporting Information Fig. 3). Of these, 39 were upregulated and two downregulated when comparing the malignant and healthy groups. Ten features were upregulated when comparing the malignant and benign groups, with five features overlapping. Across two 2D‐DIGE experiments examining two sets of separately MARS‐immunodepleted samples, 993 and 1268 matched features were detected, with more lower abundance features apparent than in the unfractionated sera (Supporting Information Fig. 3). Quantitative analysis showed 61 spots displaying differential abundance between the malignant and healthy groups and 58 between the malignant and benign groups, with 47 overlapping. For the Proteominer‐enriched samples, 967 features were detected, and again, lower abundance features were apparent. Forty‐five spots displayed differential abundance between the malignant and healthy groups and 27 between the malignant and benign groups, with 11 overlapping.

Differentially abundant protein spots (*n* = 196 with ≥1.5 average‐fold change and *p* < 0.01) were targeted for MS‐based protein identification that gave a total of 178 confident identifications, representing 56 unique gene products (see Supporting Information Table 2). As expected, multiple isoforms of some proteins were detected in different spots, while numerous spots contained more than one protein, making it difficult to attribute abundance changes to a specific protein. The proteins identified were mainly abundant serum proteins, including known acute phase reactants such as AHSG, HP, ITIH4, SERPINA1, and SERPINA3 (upregulated in malignant), immunoglobulins (generally upregulated in malignant), complement components (mixed regulation), coagulation proteins (mixed regulation), apolipoproteins, and other transport proteins (generally downregulated in malignant). Several of these had been targeted for immunodepletion using MARS, indicating that depletion was not complete and signifying that caution should be taken in selecting such candidates for further verification. Notably, several cellular proteins were identified as differentially expressed that are not normally observed in serum using gel‐based profiling approaches. These included CCDC88C, KIF15, PCNT, PITPNA, PPL, RAB2B, RALGAPA1, TBX3, and VCL. However, several of these were colocalized with other proteins in the same spot. The most promising candidates that were up‐ or downregulated in cancer versus both the benign and healthy groups and were identified as the only protein in at least one gel spot were A2M, APOA4, C4A, CFB, CFHR2, FGG, HP, HPR, PITPNA, SERPINA1, SERPINC1, TF, and GC/VDBP.

### 3.2 SELDI‐TOF profiling for identification of candidate markers

Multiple fractions of 131 individual serum samples in triplicate were generated using the Deep Proteome strategy and the fractions profiled using high‐throughput SELDI‐TOF MS, generating a total of 1786 aligned peak clusters across the whole dataset. Using data from 5 runs of control serum, the average CV for 33 randomly selected peaks from one fraction run on a CM10 chip was 16.4% ± 6.8%, demonstrating acceptable workflow reproducibility. For all peaks across all fractions and chip types, the average CV was 30% ± 10.5%, with the IMAC30 chips giving greater variance. Average peak intensities were compared between clinical groups to define potential markers with a total of 528 differences (*p* < 0.05) identified from pairwise comparisons of malignant, benign, healthy, early‐stage malignant, late‐stage malignant, healthy plus benign, and between bioprocessors (see Supporting Information Table 3.1). The data were filtered using a conservative Bonferroni‐corrected *p* value cut‐off (*p* < 0.000041) and a ROC curve AUC cut‐off (<0.2 or >0.8) to define 88 discriminatory peaks (see Supporting Information Table 3.2). These peaks were also used in a multivariate analysis using classification trees to build models that could discriminate the clinical groups and to aid in peak prioritization. The best model (a combination of peaks S‐13070, E‐4629, E‐5066, E‐43409, and S‐29201) gave 82% sensitivity and 91% specificity in discriminating M and B. Peaks were then prioritized according to their ability to discriminate early‐stage cancers and also to differentiate from the benign group.

Identities were ascribed to the peaks following MS/MS‐based analysis and from previously published work (see references in Table 2), and were also informed by a correlation analysis examining coregulation. All but ten of the 88 peaks were confidently or putatively identified as abundant serum proteins and modified forms thereof. Modified forms included different proteolytic products, PTMs, oligomeric forms, different charge states, and adducts (see Table 2 and Supporting Information Table 3.3). The proteins were derived from 17 gene products. Apolipoproteins APOA1, APOA2, and APOA4 were found to be downregulated in the malignant group, as were A2M, TF (transferrin), and TTR (transthyretin), while the known acute phase reactants A1AT/SERPINA1, SERPINC1, SAA1, SAA2, and SAA4 were upregulated, as were B2M, VDBP, IGFBP4, RNASE4, and SLPI.

**Table 2:** SELDI‐TOF peak characteristics, prioritization, and identification

Ave. *m/z*	Priority	Protein ID	Gene symbol	Refs.	Ratio: M v B	Ratio: M v H	Ratio: M v H+B	Ratio: M‐early v H	Ratio: M‐late v H
2911	C	Unknown			3.39	4.07	3.75	1.14	5.25
3446	A	Unknown			0.70	0.71	0.70	0.86	0.66
4465	C	**Antichymotrypsin, CT**	SERPINA3	37, 38	2.45	2.79	2.63	1.13	3.41
4630	C	**Antichymotrypsin, CT**	SERPINA3	37, 38	1.73	1.94	1.85	1.30	2.15
5066	A	Alpha‐1‐antitrypsin, CT?	SERPINA1		1.80	1.96	1.89	1.52	2.18
5960		**B2M 2+**	B2M	39	1.31	1.75	1.53	1.42	1.90
5998		**Fibrinogen α fragment**	FGA		1.03	1.86	1.39	2.11	1.76
8679	B	**ApoAII monomer**	APOA2	40	0.71	0.64	0.66	0.89	0.57
11 240	C	SAA2 ‐4aa	SAA2	41, 42	2.66	4.91	3.64	1.48	6.04
11 372	C	IGFBP4 CT half	IGFBP4		2.45	2.87	2.68	1.03	3.33
11 408	C	**SAA2 ‐2aa**	SAA2	41, 42	4.46	7.98	6.02	1.64	9.28
11 462		**SAA2 ‐1aa**	SAA2	41, 42	7.20	20.36	11.61	0.83	24.93
11 465	C	**SAA1 ‐2aa**	SAA1	41, 42, 43	8.53	22.21	13.36	0.98	28.66
11 505	C	**SAA2 ‐1aa**	SAA2	41, 42	6.53	22.66	11.22	1.06	27.91
11 534	C	**IGFBP4 CT half**	IGFBP4		2.33	2.86	2.61	1.20	3.39
11 548	C	**SAA1 ‐1aa**	SAA1	41, 42, 43	3.58	8.39	5.40	1.01	10.28
11 574	C	**SAA1 ‐1aa**	SAA1	41, 42, 43	4.96	7.20	6.07	1.42	7.74
11 662		**SAA2**	SAA2	41, 42	5.98	11.45	8.31	1.06	13.27
11 674	C	**SAA2**	SAA2	41, 42	5.88	9.77	7.68	1.01	11.37
11 696		**SAA1**	SAA1	41, 42, 43	7.92	21.52	12.59	0.93	24.49
11 701	C	Antileukoproteinase	SLPI		1.88	1.86	1.87	1.14	2.17
11 702	C	**SAA1**	SAA1	41, 42, 43	13.11	17.50	15.36	1.25	19.81
11 704		**SAA1 ‐1aa**	SAA1	41, 42, 43	7.65	15.64	10.93	1.17	18.92
11 709	C	Antileukoproteinase	SLPI		2.04	1.85	1.93	0.78	2.25
11 714		**SAA1**	SAA1	41, 42, 43	4.13	6.81	5.37	0.96	8.60
11 715		**SAA1 ‐1aa**	SAA1	41, 42, 43	5.24	7.78	6.48	0.98	10.23
11 722	C	**SAA1**	SAA1	41, 42, 43	3.51	4.45	4.00	0.94	5.56
11 723		**SAA1**	SAA1	41, 42, 43	4.55	5.47	5.05	1.01	5.50
11 724	C	**Antileukoproteinase**	SLPI		2.22	2.75	2.50	1.03	3.08
11 725		**SAA1**	SAA1	41, 42, 43	3.33	3.90	3.65	0.97	4.68
11 754	C	**Antileukoproteinase**	SLPI		3.19	3.60	3.42	1.18	3.98
11 864		**SAA1**	SAA1	41, 42, 43	5.50	9.43	7.28	1.48	10.68
11 909		**B2M**	B2M	39	2.13	2.89	2.52	1.68	3.25
11 921	C	**B2M**	B2M	39	1.78	2.06	1.93	1.19	2.39
12 035	C	SAA1 adduct?	SAA1	41, 42, 43	3.58	5.55	4.52	1.47	6.30
12 087	C	SAA1 SPA adduct	SAA1	41, 42, 43	2.86	4.26	3.54	1.48	4.84
12 111	C	B2M SPA adduct	B2M	39	1.85	2.34	2.11	1.12	2.77
12 548	C	Unknown			2.75	3.26	3.03	1.16	3.64
12 891	C	**SAA4**	SAA4	40	1.97	2.18	2.09	1.18	2.45
12 937	B	Unknown			1.22	1.84	1.52	1.43	1.98
13 070	A	SAA4	SAA4	40	2.05	3.06	2.54	1.81	3.63
13 739	C	TTR, ammonia loss	TTR	44, 45, 46	0.66	0.65	0.65	0.93	0.56
13 757	C	**TTR**	TTR	44, 45, 46	0.62	0.62	0.62	0.97	0.49
13 866	C	**RNAse 4**	RNASE4		1.51	1.36	1.42	1.13	1.45
13 961	A	TTR, SPA adduct	TTR	44, 45, 46	0.70	0.67	0.68	0.81	0.61
13 986	A	TTR, SPA adduct	TTR	44, 45, 46	0.70	0.64	0.66	0.80	0.58
14 056	B	TTR, glutathionylated	TTR	44, 45, 46	0.72	0.62	0.66	0.79	0.56
14 083	B	TTR, cysteinylated, SPA adduct	TTR	44, 45, 46	0.74	0.66	0.69	0.83	0.60
14 269		**ApoAI, 2+**	APOA1	40, 46, 47, 48	0.88	0.71	0.77	0.89	0.67
14 350		**ApoAI, 2+**	APOA1	40, 46, 47, 48	0.85	0.72	0.77	0.89	0.69
14 589		**ApoAI, 2+**	APOA1	40, 46, 47, 48	0.83	0.72	0.76	0.89	0.68
17 095	A	**ApoAII dimer ‐2 Gln**	APOA2		0.62	0.55	0.58	0.69	0.52
17 230	A	**ApoAII dimer ‐Gln**	APOA2		0.63	0.58	0.60	0.77	0.52
18 001	A	ApoAII dimer (+propeptide?)	APOA2		0.66	0.62	0.64	0.73	0.59
21 497		ApoAIV 2+	APOA4		0.79	0.64	0.70	0.64	0.65
21 682		ApoAIV 2+	APOA4		0.67	0.55	0.59	0.62	0.54
21 714		ApoAIV 2+	APOA4		0.72	0.58	0.63	0.56	0.60
21 808		ApoAIV 2+	APOA4		0.77	0.64	0.69	0.57	0.67
25 621	A	VDBP, 2+	VDBP		1.63	1.75	1.70	1.38	1.80
28 559	B	**ApoAI**	APOA1	40, 46, 47, 48	0.87	0.71	0.77	0.86	0.67
28 697	B	**ApoAI**	APOA1	40, 46, 47, 48	0.86	0.72	0.77	0.87	0.69
29 201	B	**ApoAI**	APOA1	40, 46, 47, 48	0.85	0.72	0.77	0.84	0.70
29 394	B	**ApoAI**	APOA1	40, 46, 47, 48	0.85	0.73	0.78	0.84	0.71
35 763	A	ApoAI + ApoAIV, 2+	APOA1		0.80	0.70	0.74	0.63	0.73
39 411	C	Unknown			2.01	2.28	2.16	1.05	2.57
40 617		Unknown			3.46	5.30	4.35	1.25	6.25
41 956	B	ApoAI + TT cysteinylated	APOA1		0.61	0.52	0.56	0.78	0.44
**43 409**	A	ApoAIV	APOA4		0.68	0.55	0.60	0.57	0.55
43 429		**ApoAIV**	APOA4		0.72	0.56	0.61	0.60	0.55
44 662	A	ApoAIV	APOA4		0.84	0.72	0.77	0.65	0.75
47 287		ApoAI + albumin, 2+	APOA1		0.72	0.60	0.64	0.83	0.53
50 764	A	**Alpha‐1‐antitrypsin**	SERPINA1		1.59	1.60	1.59	1.19	1.69
51 213	A	**VDBP**	VDBP		1.63	1.73	1.69	1.27	1.83
53 296	C	related to VDBP			1.89	2.22	2.07	1.52	2.38
56 481		**ApoAI dimer**	APOA1	40, 46, 47, 48	0.82	0.65	0.71	0.76	0.63
75 809	C	Unknown			0.76	0.71	0.73	1.00	0.61
79 221		**Transferrin**	TF	46, 49	0.84	0.83	0.83	1.03	0.76
80 641	C	Transferrin	TF	46, 49	0.74	0.65	0.68	0.96	0.53
86 919		**ApoAI trimer**	APOA1	40, 46, 47, 48	0.78	0.56	0.63	0.58	0.57
94 544	B	ApoAI + albumin	APOA1		0.73	0.62	0.67	0.80	0.56
101 711	A	**Alpha‐1‐antitrypsin dimer**	SERPINA1		1.38	1.52	1.45	1.22	1.56
116 137		**ApoAI tetramer**	APOA1	40, 46, 47, 48	0.74	0.50	0.58	0.48	0.54
117 380	B	VDBP + albumin	VDBP		2.05	2.41	2.24	1.39	2.67
123 153	C	Unknown			0.71	0.67	0.68	0.88	0.60
175 448	B	Alpha‐2‐macroglobulin	A2M		0.59	0.51	0.54	0.78	0.43
184 620	B	VDBP + albumin dimer	VDBP		2.41	3.40	2.91	1.94	3.66
264 556	C	Unknown			0.63	0.66	0.64	1.05	0.53
295 826	B	Unknown			0.68	0.58	0.62	0.65	0.56

Average *m/z* values shaded gray were peaks used in classification tree models. Priority column corresponds to the following definition: A = peak intensity different in early stage and also differentiates from benign; B  = peak intensity different in early stage and does not differentiate from benign; C = peak intensity different only in late stage. For Protein ID; bold versus plain font = increased confidence in ID; gray shading = ID based on experimental data and known modifications/dimer status; plain font and no shading = prediction only or inference from literature. Abbreviations: aa, amino acid; Apo, apolipoprotein; B2M, beta‐2‐microglobulin; CT, C‐terminal; SAA, serum amyloid A; SPA, sinapinic acid; TTR, transthyretin; VDBP, vitamin D binding protein. M, malignant; B, benign; H, healthy. At least one ratio in the five comparisons was significant (*p* < 0.000041) and had an area under the ROC curve <0.2 or >0.8. See, Supporting Information Table 3 for more detail.

### 3.3 Initial validation of candidate markers for differential diagnosis of ovarian cancer

**Figure 1:**
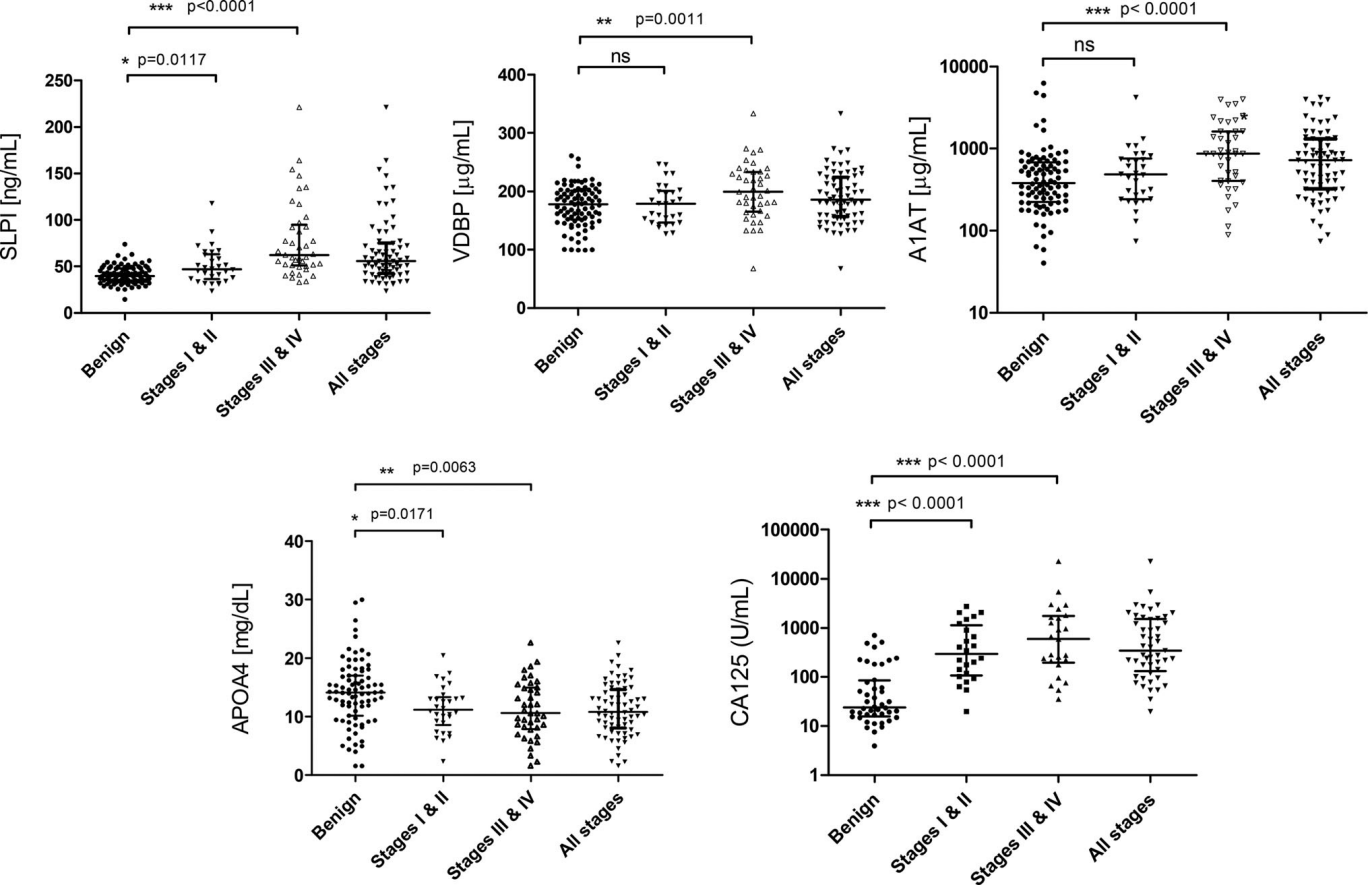
Scatter plots of serum assay values for SLPI, VDBP, A1AT/SERPINA1, APOA4, and CA125 in a set of 89 benign and 70 malignant ovarian cancer cases separated by FIGO stage (I + II and III + IV). Bars indicate the median value with interquartile range. For normally distributed data, the Student *t* test was used to assess significance of differences; otherwise the Mann–Whitney *U*‐test was used.

Candidates APOA4, VDBP, A1AT/SERPINA1, and SLPI were selected for initial validation and were measured by ELISA in serum samples from 89 benign and 70 malignant ovarian cancer cases, with the latter grouped into early‐stage (FIGO I and II) and late‐stage (FIGO III and IV) cancers. Thus, samples from 44 and 48 independent benign and malignant cases, respectively, were analyzed in addition to those used for discovery experiments. All candidate markers were able to significantly discriminate late‐stage malignant cases from benign controls in agreement with the discovery data (Fig. 1). Only SLPI and APOA4 discriminated early‐stage malignant cases from benign cases, while SLPI and A1AT also separated early from late‐stage disease (*p* = 0.0007 and *p* = 0.0031, respectively). None of the candidate markers was as good as CA125 in discriminating malignant from benign cases (Fig. 1).

Candidate markers were next combined using multivariate logistic regression and ROC curves constructed to assess the value of combined marker panels for the differential diagnosis of ovarian cancer (Table 3). No combination of markers was better than CA125 alone for discriminating the malignant versus benign groups that had an AUC of 0.884, although the best model without CA125 (combining VDBP, APOA4, and SLPI) did have a respectable AUC of 0.834. Notably, most combined models were better than CA125 alone at discriminating early from late‐stage cancers with a model that combined CA125, VDBP, and A1AT giving an AUC of 0.806.

**Table 3:** Performance of combined marker models for differential diagnosis

Model No.	No. markers	Marker 1	Marker 2	Marker 3	Marker 4	Marker 5	AUC M versus B	AUC Early versus late
28	1	VDBP					0.598	0.659
29	1	APOA4					0.661	0.517
30	1	A1AT					0.661	0.701
22	2	VDBP	A1AT				0.665	0.759
24	2	APOA4	A1AT				0.68	0.716
21	2	VDBP	APOA4				0.704	0.677
13	3	VDBP	APOA4	A1AT			0.718	0.758
15	3	VDBP	A1AT	SLPI			0.755	0.767
23	2	VDBP	SLPI				0.758	0.738
26	2	A1AT	SLPI				0.758	0.72
31	1	SLPI					0.763	0.704
16	3	APOA4	A1AT	SLPI			0.824	0.717
6	4	VDBP	APOA4	A1AT	SLPI		0.825	0.764
25	2	APOA4	SLPI				0.831	0.7
14	3	VDBP	APOA4	SLPI			0.834	0.748
4	4	CA125	VDBP	A1AT	SLPI		0.856	0.792
20	2	CA125	SLPI				0.856	0.721
12	3	CA125	A1AT	SLPI			0.857	0.752
9	3	CA125	VDBP	SLPI			0.858	0.765
8	3	CA125	VDBP	A1AT			0.862	0.806
17	2	CA125	VDBP				0.864	0.727
2	4	CA125	VDBP	APOA4	A1AT		0.866	0.802
10	3	CA125	APOA4	A1AT			0.867	0.756
7	3	CA125	VDBP	APOA4			0.869	0.737
18	2	CA125	APOA4				0.869	0.65
1	5	CA125	VDBP	APOA4	A1AT	SLPI	0.871	0.782
5	4	CA125	APOA4	A1AT	SLPI		0.872	0.762
11	3	CA125	APOA4	SLPI			0.873	0.716
3	4	CA125	VDBP	APOA4	SLPI		0.877	0.769
19	2	CA125	A1AT				0.88	0.733
27	1	CA125					0.884	0.641

Models were constructed and tested using multivariate logistic regression in Stata software. Areas under the ROC curves (AUC) are shown for all possible combinations of markers and shown in ascending order for comparison of malignant versus benign cases. Model performance for early versus late‐stage disease is also shown.

### 3.4 Testing SLPI as an early marker of ovarian cancer

SLPI, the best performing candidate in the clinical cases was further tested as an early marker and compared with CA125 using a nested case control set from within the UKCTOCS and comprising pairs of serum samples taken at different times prior to the diagnosis of ovarian cancer (Supporting Information Table 1). Thus, 148 serum samples came from 74 women, 19 of who were subsequently diagnosed with Type I or borderline (Type I/BL) ovarian cancer, 30 of who were subsequently diagnosed with Type II ovarian cancer and 25 noncancer controls. The samples were also grouped by time to diagnosis, with a 3–14 months (designated "late") and a >32 months (designated "early") prediagnosis group.

**Figure 2:**
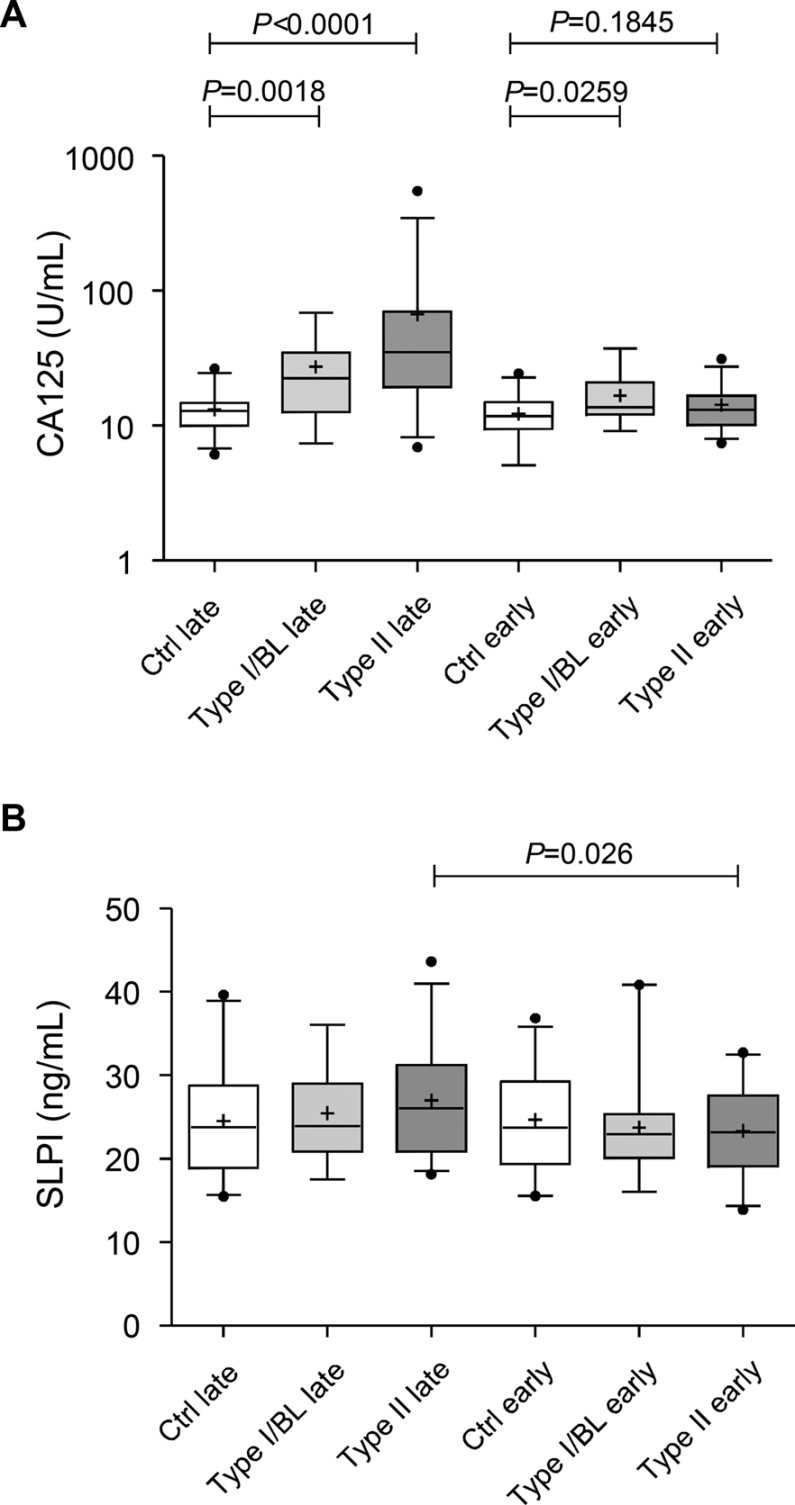
Box and whisker plots showing serum assay values for CA125 (A) and SLPI (B) in a set of 148 prediagnosis serum samples and matched noncancer controls. Cases were separated into Type I plus borderline (Type I/BL) disease and Type II disease based upon morphology and grade. Paired samples from each case were taken 2–14 months prior to diagnosis and termed "late" and >36 months prior to diagnosis and termed "early." Controls were matched based on Type II cases. Boxes indicate the median value with interquartile range and whiskers show the 5^th^ and 95^th^ percentiles. The cross indicates the mean value for each group. The Student *t* test was used to assess significance of differences for the SLPI data, which was normally distributed, while the Mann–Whitney *U*‐test was used for the CA125 data.

CA125 performed well in detecting cancers within 3–14 months of diagnosis (Fig. 2A), and was superior for the Type II cases that were predominantly high grade serous and endometrioid cancers (AUC = 0.78 for Type I/BL "late"; AUC = 0.89 for Type II "late"). As expected, AUC's for CA125 were lower for the "early" prediagnosis samples, and notably worse for Type II cases (AUC = 0.70 for Type I/BL "early"; AUC = 0.61 for Type II "early"). Serum SLPI levels did not significantly discriminate cases and controls in either prediagnosis time group, although its levels were significantly increased in the "late" versus "early" time group samples for the Type II cases (Fig. 2B). Combining CA125 and SLPI showed no improvement in the ability to detect preclinical cancer with or without additional epidemiological and lifestyle factors (not shown).

## 4 Discussion

Two complementary protein‐profiling methods were applied to serum samples taken from patients diagnosed with malignant or benign epithelial ovarian cancer and healthy controls for the unbiased identification of candidate biomarkers for differential diagnosis of ovarian cancer. It was somewhat disappointing that mostly abundant proteins were identified as being differentially expressed between the sample groups despite the use of strategies that employed extensive fractionation and immunodepletion. This highlights the very large dynamic range and complexity of protein expression in serum. While undoubtedly improving dynamic range, it is worth mentioning the limitations of serum immunodepletion and protein equalization in proteomics discovery experiments. Particularly, both methods may potentially remove diagnostic proteins bound to abundant carrier proteins such as albumin, while equalization seems conceptually at odds with the ability to accurately compare starting protein levels across sample groups. There is the assumption that protein levels in the starting material are preserved after equalization that may not be the case. However, we do note that a subset of the same differentially expressed proteins was identified using the two different profiling methods, suggesting that such an effect is not prevalent. Despite the limited coverage, several cellular, nonsecreted proteins were identified as being differentially regulated and the phospholipid transfer protein PITPNA was the top candidate in this class for discriminating malignant and benign cases. PITPNA has not been previously reported as a cancer biomarker and warrants further investigation.

Notably, four of the differentially expressed proteins identified (namely APOA1, B2M, TF, and TTR) are the four serum proteins used with CA125 in the Ova1 diagnostic test 6, 10, 27, approved for use in combination with physician assessment to determine if an ovarian tumour on ultrasound warrants referral for surgery. These findings thus add confidence to the discovery and partly support the validity of the markers used in the Ova1 test. Whilst the performance of the non‐CA125 Ova1 markers without CA125 has not been evaluated directly, one study showed that the Ova1 test detected over 50% of malignancies missed by CA125 alone 28. Without inclusion of physician's assessment, the sensitivity and specificity of CA125 alone (cut‐off 35 U/mL) were 77 and 73%, respectively, versus 93 and 43% using the Ova1 test. Thus, inclusion of APOA1, B2M, TF, and TTR improve the sensitivity of the test at a significant cost to specificity.

Herein, an increase in known acute‐phase proteins was observed in the malignant versus benign (and healthy) samples. This suggests that malignancy is accompanied by an increased inflammatory response versus the benign conditions. Whether this response is specific to ovarian malignancy would require further investigation, for example, comparing these candidate markers in malignant and benign ovarian cancer cases versus inflammatory disease cases such as arthritis. Similarly, there was a general decrease in proteins reflective of nutritional status such as serum apolipoproteins and other serum transport proteins. Indeed the best candidate markers for differential diagnosis of ovarian cancer identified here fell into these functional categories, including proteins A1AT, APOA4, and VDBP that were selected for further evaluation as ovarian cancer biomarkers. It may also be the case that SLPI (the fourth candidate tested) is also raised during inflammation. SLPI is expressed in a tissue‐specific manner by cells at a variety of mucosal surfaces and is an inhibitor of the proteases elastase, cathepsin G and trypsin 29. Together with A1AT it is thought to protect mucosal surfaces, particularly in the lung, from damage by neutrophil elastase 30. SLPI has been tested as an ovarian cancer biomarker previously, with reasonable accuracy for differential diagnosis of malignant disease 31. In another case‐control study, serum SLPI showed stage‐specific elevation, although was not selected for inclusion in the multimarker models that were tested 32. Our data support these previous findings and suggest that SLPI may have some utility as a serum marker for ovarian cancer, although combining SLPI with CA125 did not improve on CA125 as a single marker. Serum SLPI was also poor at predicting preclinical ovarian cancer cases, although there was evidence of a rise in levels in cases toward diagnosis. VDBP has not previously been reported as an ovarian cancer marker, although using a peptide profiling approach, endogenous proteolytic fragments of VDBP were identified in ascites fluid from ovarian cancer patients, but not from control patients with liver cirrhosis 33. The authors however reported no difference in serum VDBP protein levels (by ELISA) between the ovarian cancer cases and healthy controls. Finally, plasma APOA4 was previously reported to be lower in samples from cases of malignant versus benign ovarian neoplasms, but did not add independent diagnostic information to CA125 34. Indeed, no combination of the candidate markers reported here was better than CA125 alone at differentiating malignant from benign ovarian neoplasms. However, it is important to note that CA125 performs well in this cohort (92% sensitivity, 70% specificity at a 37 U/mL cut‐off). As we have previously reported 35, this likely reflects the fact that samples were obtained from women referred to specialist gynaecologic cancer centres, a proportion of which will have been referred based on elevated CA125. Thus, the performance of CA125 can be exaggerated in these types of study, as previously highlighted 36. The better‐than‐expected performance of CA125 may also in part be explained by the inclusion of only postmenopausal women in our cohort and restriction of cases to only primary invasive epithelial cancers.

In conclusion, we have identified and tested several candidate serum markers of ovarian cancer that are presumed indicators of inflammation and nutritional status. This suggests that there are differences in the body's response to the presence of a malignant versus benign ovarian cancer. Although the markers could accurately discriminate malignant from benign cases, none were as good as CA125 either alone or in combination having low specificities, and did not add to the performance of CA125, which was high in this cohort. Despite this, further independent validation of these candidate markers is warranted, using a more randomly selected patient cohort, for example, any woman referred based on an unusual transvaginal ultrasound result.

## Supporting information

Yes

Supplementary Data – Materials and Methods

Figure S1 HPLC chromatograph overlays. MARS‐depletion of pooled clinical sera was repeated 10 times. Representative overlays of 3 runs each for A) healthy, B) benign and C) malignant late stage groups are shown.
Figure S2 1D‐SDS‐PAGE comparison of ProteoMiner‐ fractionated samples. 25 μg of protein from unfractionated (U), flow‐through (FT) and bound (B) fractions for each clinical condition were run on a 12% SDS PAGE gel which was then stained with CCB.
Figure S3 Representative fluorescence 2D gel images of unfractionated, MARS fractionated and Proteominer fractionated sera

Table S1 Characteristics of prediagnosis sample set. The study set comprised serum from women in the multimodal screening arm of UKCTOCS. There were two samples from each of 49 women taken 3–14 months (‘late’) and >32 months (‘early’) prior to diagnosis of primary invasive or borderline epithelial ovarian cancer. These were subsequently grouped as Type I + Borderline (BL) and Type II, based on morphology and grade. Matched non‐cancer controls from 25 women (2 samples each) were selected based on collection date, collection centre and age for the Type II cases. The resulting study set was 148 serum samples from 74 women, 19 of whom were subsequently diagnosed with Type I or BL ovarian cancer and 30 of whom were subsequently diagnosed with Type II ovarian cancer.

Table S2 MS‐based protein identifications from 2D‐DIGE profiling of unfactionated, MARS‐depleted and Proteominer‐equalised serum pools. Serum from 131 cases and controls were pooled according to clinical group (M = malignant; B = benign; H = healthy). Pools were left unfractionated (UnF), MARS‐depleted in two separate experiments (MARS1 and 2) or subjected to Proteominer equalisation (PMF) and the samples compared in quadruplicate by 2D‐DIGE using a mixture of all samples as an internal standard. Spots displaying a >1.5‐fold difference in abundance (P <0.05) were targeted for spot picking, tryptic digestion and MS‐based identification using MALDI‐TOF MS (UnF and MARS1) or LC‐MS/MS (MARS2 and PM). Spot number, protein name, IPI accession number, fuctional class, MASCOT score, sequence coverage, number of unique peptides, predicted molecular weight, pI and abundance changes and P values across clinical groups are given. In cases where multiple identifications were made from the same gel spots, all protein groups are reported

Supplementary Data ‐ Table S3 ‐ SELDI profiling
