# Supplementary material for: Discovery of serum biomarkers of ovarian cancer using complementary proteomic profiling strategies
Source: Proteomics Clin Appl. 2014 Nov 10;8(11-12):982–93. doi: 10.1002/prca.201400063 (PMC4737403; doi:10.1002/prca.201400063)
Supplement: Supplementary file 1 — Supplementary Data – Materials and Methods [file PRCA-8-982-s001.pdf]

Supplementary Data – Materials and Methods

Sample preparation for 2D-DIGE

Unfractionated samples

Prior to 2D-DIGE analysis, unfractionated serum samples were pooled according to volume into healthy, benign or malignant groups. Protein concentrations were determined using the Pierce BCA protein assay and BSA to generate a standard curve. Samples were then diluted to the same protein concentration of 0.8 mg/mL (~100-fold dilution) with 2D lysis buffer (8 M urea, 2 M thiourea, 4% CHAPS, 0.5% NP40 and 10 mM Tris pH 8.3) prior to 2D-DIGE.

Immunodepletion

Pooled samples were immunodepleted of the top 7 most abundant proteins prior to 2D-DIGE profiling using the HPLC-based Multiple Affinity Removal System (MARS). This is comprised of an affinity HPLC column (size: 4.6 x 50 mm, Agilent part number 5185-5984) packed with immobilised affinity-purified polyclonal antibodies for removal of albumin, transferrin, IgG, IgA, haptoglobin, antitrypsin and fibrinogen with high specificity and optimised mobile phases.

All chromatographic steps were performed at 20°C on an Agilent 1100 HPLC system. From each pool, 30 µL of serum was diluted five times with MARS Buffer A containing protease inhibitors (COMPLETE™, Roche) and centrifuged at 16,000 x g at room temperature for 5 minutes to remove any particulates. Automated sample injection was set up for 30 µL of diluted serum sample per injection in Buffer A at a flow rate of 0.25 mL/min for 9 min. Flow-through fractions, ~0.75 mL per injection, containing the lower abundant protein species were collected from each injection manually at 2-4 min into 0.5 mL Eppendorf tubes and stored at -20°C until further analysis. The bound fractions were eluted with 100% Buffer B at a flow rate of 1.0 mL/min for 3.5 min. The column was regenerated by equilibrating with Buffer A for 10 min. Overlay of chromatograms from multiple runs demonstrated a high degree of reproducibility (see Supplementary data Figure S1) and similar protein yields were recorded (CV = 7%).

For protein desalting and concentration prior to 2D-DIGE, the MARS flow-through fractions from five injections were pooled into a 5 mL Zeba™ spin column (Pierce, Rockford, IL) and desalted. Desalted samples were concentrated to 0.5 mL using a spin concentrator with a 5 kDa Molecular Weight Cut-Off (MWCO) membrane; samples were spun at 4000 x g for approximately 1 hour at 10 °C. Concentrated retentates were transferred into fresh tubes and speed vacuumed to dryness. Samples were resuspended in 2D lysis buffer. Protein content was estimated using the Pierce BCA protein assay using BSA to generate a

standard curve and protein concentrations equalised to 1 mg/mL with 2D lysis buffer prior to 2D-DIGE.

ProteoMiner enrichment - Pooled samples were applied to spin columns from the ProteoMiner Protein Enrichment Kit (BioRad Catalogue # 163-3000). The ProteoMiner Protein Enrichment Kit is comprised of several spin columns packed with a large, highly diverse bead-based library of combinatorial peptide ligands. It is considered to be a novel sample preparation tool used for the compression of the dynamic range of the protein concentration in complex biological samples. Best results are obtained with protein concentrations greater than 50 mg/mL. When complex biological samples (e.g. human serum) are applied to the beads, the high abundance proteins saturate their high affinity ligands and the excess protein is washed away. In contrast, the medium and low abundance proteins are concentrated on their specific affinity ligands. This reduces the dynamic range of protein concentrations, while maintaining representatives of all proteins within the original sample.

Spin columns were prepared by centrifugation at 1,000 x g for 2 min to remove storage solution. The columns were then washed by adding 1 mL deionised water and rotating them end-to-end for 5 min. Columns were again centrifuged at 1,000 x g for 2 min to remove the water and the wash step repeated twice using 1 mL wash buffer (PBS). At the final wash step, columns were centrifuged again for an additional 1 min at 1,000 x g to remove any remaining buffer.

Pooled serum samples were centrifuged at 10,000 x g for 10 min to remove particulates. 1 mL of serum (>50 mg/mL), normalised for protein concentration, was applied to the spin columns and incubated with the beads by rotation for 2 hr at room temperature. Columns were centrifuged at 1,000 x g for 2 min and the collected material was retained for analysis. The columns were centrifuged again at 1,000 x g to remove residual material. Next, the columns were washed 3 times with 1 mL of wash buffer (PBS) each and any remaining liquid removed by a final centrifugation step. Bound proteins were eluted with 100 µL of 2D lysis buffer by gentle vortexing over a 15 min period at ambient temperature and the eluate collected by centrifugation at 1,000 x g for 2 min. The elution step was repeated twice to ensure all bound material was collected. Protein concentration of the eluates was estimated using the Pierce BCA protein assay using BSA to generate a standard curve. Recoveries were 632.4 µg total protein from the healthy pool, 687.4 µg from the benign pool and 786.8 µg from the malignant pool. Sample concentrations were equalised to 2 mg/mL with 2D lysis buffer and the samples stored at -20°C prior to downstream 2D-DIGE analysis. The bound and flow-through fractions were also separated by 1D-SDS-PAGE alongside unfractionated sera to assess the degree of enrichment/equalization (Supplementary Data; Figure S2).

2D-DIGE profiling

The three sample preparations were labelled with Cy-dyes and run in a series of 2D-DIGE experiments to compare the proteomes of the healthy, benign and malignant serum pools, essentially as described [1] (see also Supplementary Data for details). Samples (50 or 100 μg) were run in quadruplicate with Cy3 and Cy5 dye swapping against an internal standard run on each gel comprising of a Cy2-labelled pool of the clinical groups for the relevant sample preparation method.

NHS-cyanine dye Cy2 was purchased from GE Healthcare, whilst NHS-Cy3 and NHS-Cy5 were synthesised "in-house"[2]. Samples were labelled with Cy-dye at 4 pmol dye/µg protein on ice in the dark for 30 min. Labelling reactions were quenched with a 20-fold molar excess of free L-lysine to dye and left on ice for 10 min. Equal amounts of proteins labelled with Cy3 and Cy5 were mixed appropriately and the same amount of Cy2-labelled pool was added to each mixture. Samples were reduced by adding 1.3 M dithiothreitol (DTT) to a final concentration of 65 mM. Ampholine/Pharmalyte carriers (1:1 mix, pH 3-10), were added to a final concentration of 2% and bromophenol blue (0.01%) was added to each sample. The final volume of each sample was adjusted to 450 µL with 2D-DIGE lysis buffer plus DTT. For isoelectric focusing (IEF), 24 cm, non-linear pH 3-10 IPG strips (GE Healthcare) were rehydrated with Cy-dye labelled samples in a re-swelling tray overnight in the dark at RT overlaid with mineral oil. IEF was carried out on a Multiphor II apparatus (GE Healthcare) for a total of 80 kVh at 15°C.

For protein separation in the second dimension, 1.0 mm 12% SDS-PAGE gels were cast between 24 cm low-fluorescence glass plates. The inner surface of one plate of each set was coated with Bind Silane solution (PlusOne, GE HealthCare) to bond the gels. This allows easier handling of gels during scanning and protein post-staining, storage and spot excision. Fluorescent reference markers were placed at the edges of the bonded plates for generation of coordinates for robotic picking. The inner surface of the other plate was treated with Repel Silane (PlusOne, GE Healthcare) to ensure easy separation of plates after running. After IEF, IPG strips were equilibrated in equilibration buffer (6 M urea, 30 % (v/v) glycerol, 50 mM Tris-HCL pH 6.8 and 2% (w/v) SDS) in two steps for 15 minutes each with gentle rocking. In the first step, the equilibration buffer was supplemented with 65 mM DTT to reduce disulphide bonds, while in the second step 240 mM iodoacetamide (IAM) was added to the equilibration buffer to alkylate reduced thiol groups. IPG strips were then rinsed with Tris-Glycine-SDS electrophoresis buffer (Severn Biotech) and transferred onto the second dimension gels. Strips were overlaid with 0.5% (w/v) low-melting point agarose in Tris-Glycine-SDS electrophoresis buffer with bromphenol blue. Gels were run in an Ettan 12 apparatus (GE Healthcare) at 2.2 W per gel at 16°C until the dye front had run off, thereby

avoiding the fluorescence signal from bromophenol blue and free dye. All steps were carried out in a dedicated clean room.

Gel images were obtained by scanning the gels between plates on a Typhoon™ 9400 multi-wavelength fluorescence scanner using ImageQuant software (both from GE Healthcare). The photomultiplier tube voltage of the Typhoon scanner was adjusted for each channel (Cy2, Cy3, and Cy5) in preliminary low-resolution scans (1000 µm) to give maximum pixel values within 10% for each channel, but below the saturation level. These settings were then used for high-resolution (100 µm) scanning. Images were generated as .gel/TIFF files and exported to DeCyder image analysis software for analysis of differential expression.

Gel images were analysed using DeCyder™ image analysis software V5.0 (GE Healthcare). Firstly, images were analysed using the Differential In-Gel Analysis (DIA) module for automatic normalisation, spot detection, filtering and background subtraction and quantification of protein spot abundance/volume on each image. Subsequently, the Biological Variance Analysis (BVA) module was used for matching protein spots from different conditions across gels by matching to a user defined master gel image. User intervention was required at this stage to set landmarks on gels for accurate cross-gel matching. Standardised spot volumes were then averaged across replicate samples for each experimental condition and data plotted graphically within BVA. Statistical analysis was performed and spots matched on all gels displaying a ≥1.5 average-fold increase or decrease in abundance between clinical conditions with *P* values <0.01 (Student t-test) were selected for spot picking and MS-based identification.

Bonded 2D gels were post-electrophoretically stained with colloidal Coomassie Blue G-250 (CCB) to visualise proteins for accurate spot picking. Gels were stained according to a modified protocol by Neuhoff et al.[3] Briefly, bonded gels were fixed in 35% (v/v) ethanol with 2% (v/v) phosphoric acid for more than 3 hours on a shaking platform and then washed three times for 30 min each in ddH2O. Gels were then incubated in 34% (v/v) methanol, 17% ammonium sulphate and 3% (v/v) phosphoric acid for one hour prior to addition of 0.5 g/L Coomassie Blue G-250 (Merck Biosciences) and left to stain for two to three days. De-staining was not required.

Post-stained gels were scanned on the Typhoon™ 9400 scanner using the red laser with no emission filter. Post-stained images were imported into the BVA module of DeCyder and matched with the processed Cy-Dye images. Using the reference markers fixed onto the glass plates during gel casting, a pick list of coordinates (.txt file) for protein features that were differentially expressed was created for automated spot picking. An Ettan automated spot picker (GE Healthcare) was used with a 2 mm picking head, which excised protein

features from gels submerged under 1-2 mm of ddH2O. Spots were collected in 96-well plates, drained and stored at -20°C prior to MS analysis.

SELDI-TOF Profiling

The second profiling method used a proprietary, automated, multi-dimensional fractionation strategy (Deep Proteome; Ciphergen Biosystems) run on ProteinChip Arrays and coupled to high-throughput SELDI-TOF MS profiling. The method was applied to 131 individual samples (64 healthy, 45 benign and 22 malignant) run in triplicate and randomised across plates. Fractionation steps were performed in 96-well filter plates on a vacuum manifold and with liquid handling using a Tecan Aquarius robot with tip-loader. A control serum sample (Intergen) was also run (n=5) in order to monitor reproducibility.

In detail, 200 μL of neat serum was denatured with 50 μL of 9M urea/2 M guanidine thiocyanate/2% CHAPS/1% Triton X100 (TX100), treated with protease inhibitors and incubated with 35 μL Protein Equalizer Beads™ in 96-well filter plates (Nunc). The flow-through was collected on a vacuum manifold, and then three elutions steps of 100 μL each applied: 1 M NaCl (E1), 25% isopropanol (IPrOH)/25% acetonitrile (ACN)/1% formic acid (FA)/2% trifluoroacetic acid (TFA) (E2), and 8 M guanidine hydrochloride (GnHCl)/1% TX100 followed by 6 M urea/30% ethylene glycol/25 mM sodium carbonate pH 10.0 (E3). The flow-through was then applied to 100 μL of MEP (4-mercapto-ethyl-pyridine) HyperCel resin in 96-well filter plates for hydrophobic charge induction chromatography. The FT fraction from this step was collected and retained for analysis (FT) and then three elution steps of 150 μL each applied: 37.5% IPrOH/37.5% ACN/0.5% FA/1% TFA (M1), 8 M urea/5% acetic acid pH 3.0 (M2), and 8 M GnHCl/3 M Urea/1% TX100 (M3). Fractions M1 and M2 were pooled and 300 μL of 25 mM sodium acetate (NaOAc)/0.1% octyl-β-D-glucopyranoside (OGP) was added. This mixture was then added to 75 μL of ceramic S HyperD F beads in 96-well filter plates for strong cation exchange chromatography. The FT from this step was collected and the following elution steps applied: 200 μL of 1M urea/2% CHAPS/25 mM NaOAc pH 4.0 (S1), two times 100 μL of 1M urea/0.15 M NaCl/50 mM potassium phosphate pH 6.5/0.1% OGP (S2), two times 100 μL of 1 M urea/1 M NaCl/TrisHCl pH 8.0/0.1% OGP (S3), and 120 μL 8M GnHCl/1%TX100, followed by 100 μL of 6 M urea/30% ethylene glycol/25 mM sodium carbonate pH 10.0 (combined to give S4). The flow-through from the S HyperD F was pooled with fraction S1. Unfractionated and denatured serum was also prepared for analysis: 50 μL of 0.6% SDS was added to 10 μL of neat serum and the mixture heated at 90°C for 30 min (SDS). Thus, 10 fractions (E1, E2, E3, M3, S1, S2, S3, S4, FT and SDS) were prepared in triplicate for each sample.

For SELDI-TOF analysis, fractions E1, E2, S1, S2, S3, FT and SDS were applied to equilibrated CM10 (weak cation-exchange) protein array chips after dilution in binding buffer A (0.1% OGP/25 mM NaOAc pH 5.0): 20 μL of fraction E1 and FT and 10 μL of fraction E2, S1, S2, S3 and SDS were diluted with 80 μL and 90 μL of binding buffer A, respectively. CM10 chip equilibration involved two incubations with 100 μL of binding buffer A for 5 min each. Samples were allowed to bind for 1 hour and then chips were washed twice with 100 μL each of binding buffer A with repeated dispensing and aspiration (10 times). Chip arrays were then rinsed once with 100 μL of water and then air dried. All steps were performed on a Tecan Aquarius liquid handling robot.

Fractions E3, M3, S4, FT and SDS were applied to 'pre-activated' IMAC30-Cu^{2+} (immobilized metal affinity capture) chips following dilution in binding buffer B (0.1%OGP 0.1 M Tris-HCl pH 7.5): 30 μL of fraction E3, M3, S4 and FT and 10 μL of fraction SDS were diluted with 70 μL and 90 μL of binding buffer B, respectively. IMAC30-Cu^{2+} chip pre-activation involved incubation with 100 μL of 100 mM $CuSO_4$ for 10 min, followed by washing in 100 μL of water and then two incubations in binding buffer B for 5 min each. Samples were allowed to bind for 1 hour and then chips washed twice with 100 μL each of 1 M urea/2% CHAPS/0.3 M KCl/0.1 M Tris-HCl pH 7.5 with repeated dispensing and aspiration (10 times). Chip arrays were then rinsed once with 100 μL of water and allowed to air dry. All steps were performed on a Tecan Aquarius liquid handling robot.

Following sample application and air drying, two times 1 μL of matrix solution (20 mg/mL sinapinic acid/50% ACN/0.5% TFA) was applied, and the spots were air dried for 10 min. Protein arrays were then read on a calibrated PCS4000 instrument (Ciphergen Biosystems) with automated chip loading, sample tracking and chip reading controlled by Ciphergen Express software. Calibrations were made using all-in-one protein and peptide calibrants at appropriate focus masses. A 3500 nJ laser energy was used for both chip types with different mass ranges and focus masses applied depending on chip and fraction type (see below). Acquisition settings were 500 Da for matrix attenuation, 800 mHz for sampling rate, 600 shots per spot with 2 warming shots and 10 data shots per position.

For CM10 profiled FT, E1 and E2 fractions, three readings were taken: a low mass range (3-30 kDa) with focus mass at 8 kDa and two high mass range (10-350 kDa) with focus mass at 18 kDa, taken before and after formic acid addition (1 μl 25% IPrOH/25% ACN/5% FA/1% TFA). For IMAC30 FT and E3, four readings were taken: two low mass range with focus masses at 4 kDa and 8 kDa, a high mass range with focus mass at 18 kDa before formic acid addition, and one at 19 kDa after formic acid addition. For SDS fractions profiled on CM10 and IMAC30 chip arrays, three readings were taken: one with focus mass at 4 kDa for both arrays and one with focus mass at 19 kDa for the CM10 array. For CM10 S1, S2

and S3 and IMAC30 M3 and S4, two readings were taken: one with focus mass at 8 kDa and one with focus mass at 19 kDa after formic acid addition. In total, 30 profiles were generated for each sample in triplicate, resulting in a total of 11,790 spectra.

Protein identification by mass spectrometry

For protein identification from 2D gels, protein spots were subjected to trypsin digestion. Gel pieces were washed three times with 50% (v/v) acetonitrile (ACN), dried in a SpeedVac for 10 min, reduced with 10 mM DTT in 5 mM ammonium bicarbonate pH 8.0 (AmBic) for 45 min at 50°C and then alkylated with 50 mM iodoacetamide (IAM) in 5 mM AmBic for one hour in the dark at RT. Gel pieces were then washed three times in 50% (v/v) ACN and vacuum-dried prior to re-swelling with 50 ng of modified trypsin (Promega) in 5 mM AmBic pH 8.0. Gel pieces were then overlaid with 10 µL of 5 mM AmBic and digested for 16 hours at 37°C. Supernatants were collected and trypsin digests were further extracted by washing the gel pieces twice with 5% (v/v) trifluoroacetic acid (TFA) in 50% ACN. Peptide extracts from each gel piece were pooled, vacuum-dried and resuspended in 5 µL of 0.1% formic acid (FA) and stored at -20°C prior to MS analysis.

Identification of differential SELDI-TOF peaks was carried out essentially as described [4]. Briefly, proteins were fractionated by LC and ultrafiltration and identified either by direct MS/MS sequencing (<5 kDa) or purified by SDS-PAGE prior to staining, excision, tryptic digestion and MS/MS (>5 kDa). One quarter of each band was extracted from gels without digestion and re-analysed by SELDI-TOF MS to confirm matching of the band with the peak of interest. Samples were analysed on a Q-STAR XL equipped with a PCI-1000 ProteinChip Interface (Ciphergen Biosystems) and data searched against the SwissProt or NCBInr databases. Peak identifications were also confirmed using data from previous publications and informed by a correlation (Spearman's rank test) analysis.

References

1. Sinclair J, Metodieva G, Dafou D, Gayther SA, Timms JF. Profiling signatures of ovarian cancer tumour suppression using 2D-DIGE and 2D-LC-MS/MS with tandem mass tagging. Journal of proteomics 2011;74:451-65.

2. Chan HL, Gharbi S, Gaffney PR, Cramer R, Waterfield MD, Timms JF. Proteomic analysis of redox- and ErbB2-dependent changes in mammary luminal epithelial cells using cysteine- and lysine-labelling two-dimensional difference gel electrophoresis. Proteomics 2005;5:2908-26.

3. Neuhoff V, Arold N, Taube D, Ehrhardt W. Improved staining of proteins in polyacrylamide gels including isoelectric focusing gels with clear background at nanogram sensitivity using Coomassie Brilliant Blue G-250 and R-250. Electrophoresis 1988;9:255-62.

4. Timms JF, Arslan-Low E, Gentry-Maharaj A, Luo Z, T'Jampens D, Podust VN, Ford J, Fung ET, Gammerman A, Jacobs I, Menon U. Preanalytic influence of sample handling on SELDI-TOF serum protein profiles. Clin Chem 2007;53:645-56.
