# Supplementary material for: Discovery of serum biomarkers of ovarian cancer using complementary proteomic profiling strategies
Source: Proteomics Clin Appl. 2014 Nov 10;8(11-12):982–93. doi: 10.1002/prca.201400063 (PMC4737403; doi:10.1002/prca.201400063)
Supplement: Supplementary file 2 — Figure S1 HPLC chromatograph overlays. MARS‐depletion of pooled clinical sera was repeated 10 times. Representative overlays of 3 runs each for A) healthy, B) benign and C) malignant late stage groups are shown. Figure S2 1D‐SDS‐PAGE comparison of ProteoMiner‐ fractionated samples. 25 μg of protein from unfractionated (U), flow‐through (FT) and bound (B) fractions for each clinical condition were run on a 12% SDS PAGE gel which was then stained with CCB. Figure S3 Representative fluorescence 2D gel images of unfractionated, MARS fractionated and Proteominer fractionated sera [file PRCA-8-982-s002.pdf]

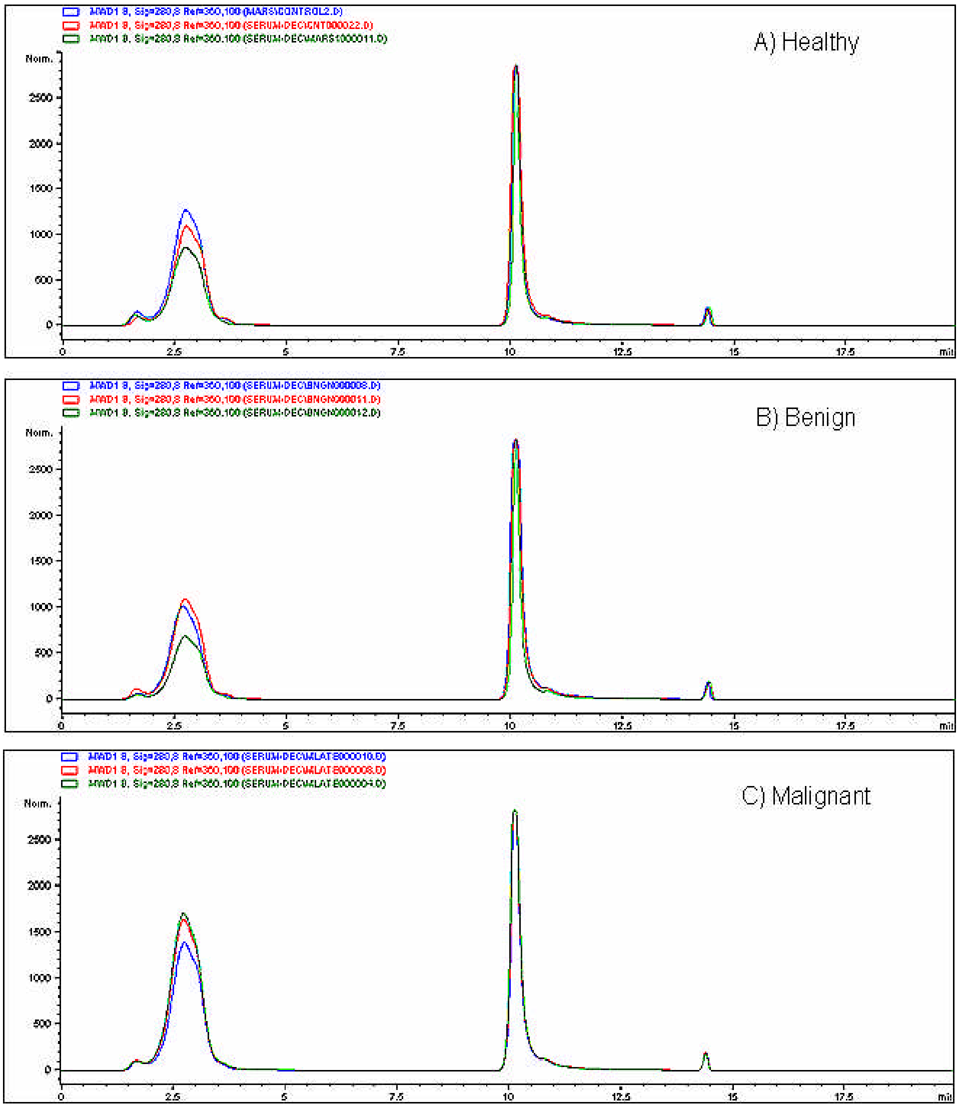

Figure S1 HPLC chromatograph overlays. MARS-depletion of pooled clinical sera was repeated 10 times. Representative overlays of 3 runs each for A) healthy, B) benign and C) malignant late stage groups are shown.

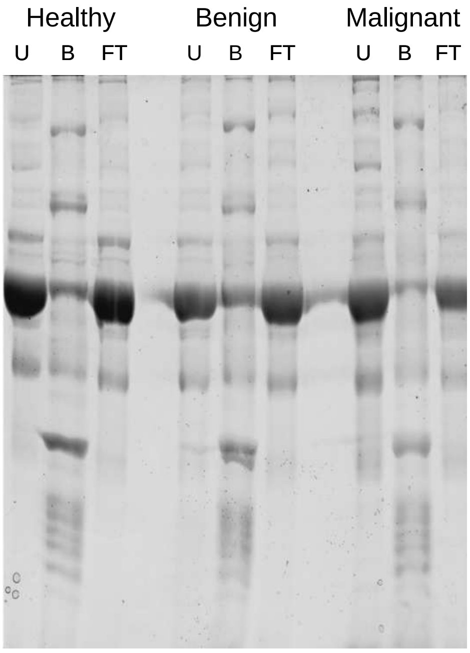

Figure S2 1D-SDS-PAGE comparison of ProteoMiner- fractionated samples. 25 µg of protein from unfractionated (U), flow-through (FT) and bound (B) fractions for each clinical condition were run on a 12% SDS PAGE gel which was then stained with CCB.

Unfractionated serum

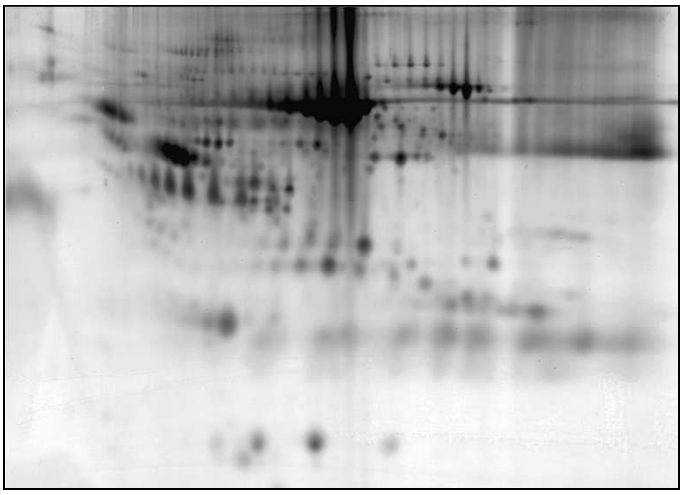

MARS fractionated

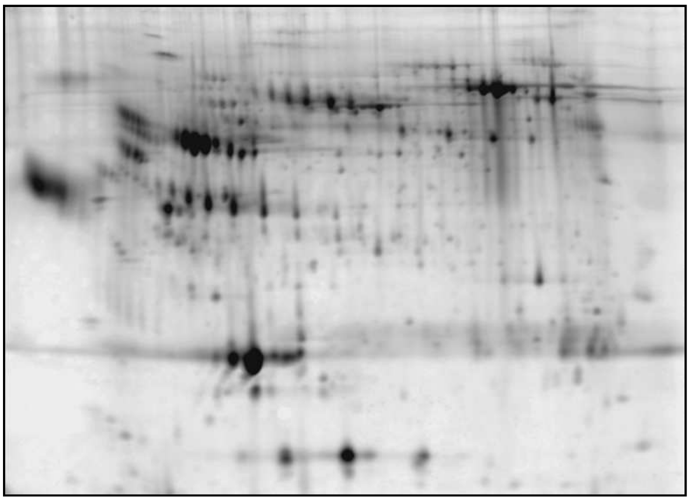

Proteominer fractionated

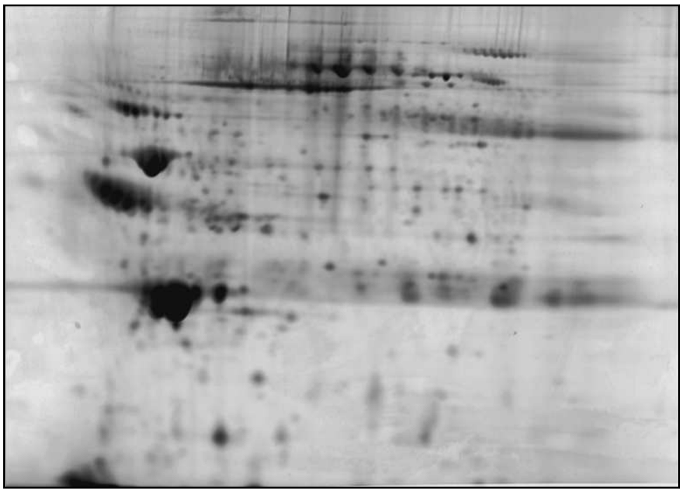

Figure S3 Representative fluorescence 2D gel images of unfractionated, MARS fractionated and Proteominer fractionated sera
