# Supplementary material for: Discovery of serum biomarkers of ovarian cancer using complementary proteomic profiling strategies
Source: Proteomics Clin Appl. 2014 Nov 10;8(11-12):982–93. doi: 10.1002/prca.201400063 (PMC4737403; doi:10.1002/prca.201400063)
Supplement: Supplementary file 3 — Table S1 Characteristics of prediagnosis sample set. The study set comprised serum from women in the multimodal screening arm of UKCTOCS. There were two samples from each of 49 women taken 3–14 months (‘late’) and >32 months (‘early’) prior to diagnosis of primary invasive or borderline epithelial ovarian cancer. These were subsequently grouped as Type I + Borderline (BL) and Type II, based on morphology and grade. Matched non‐cancer controls from 25 women (2 samples each) were selected based on collection date, collection centre and age for the Type II cases. The resulting study set was 148 serum samples from 74 women, 19 of whom were subsequently diagnosed with Type I or BL ovarian cancer and 30 of whom were subsequently diagnosed with Type II ovarian cancer. [file PRCA-8-982-s003.pdf]

Table S1 Characteristics of prediagnosis sample set. The study set comprised serum from women in the multimodal screening arm of UKCTOCS. There were two samples from each of 49 women taken 3-14 months (‘late’) and >32 months (‘early’) prior to diagnosis of primary invasive or borderline epithelial ovarian cancer. These were subsequently grouped as Type I + Borderline (BL) and Type II, based on morphology and grade. Matched non-cancer controls from 25 women (2 samples each) were selected based on collection date, collection centre and age for the Type II cases. The resulting study set was 148 serum samples from 74 women, 19 of whom were subsequently diagnosed with Type I or BL ovarian cancer and 30 of whom were subsequently diagnosed with Type II ovarian cancer.

Cancer/Control Group	Time from sample taken to Dx (months)	Time from sample taken to spin (hrs)	Histology	Cancer Morphology	Grade	Stage	HRT USE AT RECRUITMENT	HRT USE AT FOLLOW-UP	OC Pill use	Smoker	BMI	Age at sample taken	CA125 [U/mL]	SLPI [ng/mL]
Type I/BL early	84.78	24.11	Primary invasive epithelial malignant neoplasm	83803: Endometrioid carcinoma (C56)	Grade 2	Ia	YES	NO	NO	NO	30.66	68	20.96	29.22
Type I/BL early	66.75	23.92	Primary invasive epithelial malignant neoplasm	83803: Endometrioid carcinoma (C56)	Grade 2	Ia	NO	NO	YES	NO	26.44	64	25.99	19.71
Type I/BL early	78.66	17.76	Primary borderline epithelial malignant neoplasm	84423: Serous cystadenoma, borderline malignancy (C56)	Borderline	Ia	NO	NO	NO	YES	29.51	70	18.19	40.85
Type I/BL early	72.36	17.89	Primary borderline epithelial malignant neoplasm	84723: Mucinous cystadenoma, borderline malignancy (C56)	Borderline	Ic	NO	NO	NO	NO	23.30	64	15.81	20.33
Type I/BL early	74.07	20.41	Primary borderline epithelial malignant neoplasm	84623: Papillary serous cystadenoma, borderline malignancy (C56)	Borderline	Ia	NO	NO	NO	NO	23.17	73	12.21	27.15
Type I/BL early	32.48	23.09	Primary invasive epithelial malignant neoplasm	83803: Endometrioid carcinoma (C56)	Grade 2	Ic	NO	NO	YES	NO	30.42	62	12.47	31.07
Type I/BL early	55.51	23.27	Primary borderline epithelial malignant neoplasm	84423: Serous cystadenoma, borderline malignancy (C56)	Borderline	Ia	NO	NO	YES	YES	31.02	54	12.08	24.53
Type I/BL early	55.74	44.81	Primary invasive epithelial malignant neoplasm	83103: Clear cell adenocarcinoma NOS	Grade 3	IIc	NO	NO	NO	NO	23.83	74	9.80	16.86
Type I/BL early	37.6	24.41	Primary invasive epithelial malignant neoplasm	83103: Clear cell adenocarcinoma NOS	Grade 3	Ic	YES		YES	NO	28.09	60	15.18	21.19
Type I/BL early	57.83	17.42	Primary borderline epithelial malignant neoplasm	84723: Mucinous cystadenoma, borderline malignancy (C56)	Borderline	Ia	NO	NO	YES	YES	22.77	63	22.05	23.47
Type I/BL early	34.91	23.78	Primary invasive epithelial malignant neoplasm	83813: Endometrioid adenofibroma, malignant (C56)	Grade 2	IIa	NO	NO	NO	NO	30.07	60	37.28	24.10
Type I/BL early	64.08	22.74	Primary borderline epithelial malignant neoplasm	83811: Endometrioid adenofibroma, borderline malignancy (D39.1)	Borderline	Ia	NO	NO	NO	NO	32.17	66	17.71	19.17
Type I/BL early	72.73	26.8	Primary borderline epithelial malignant neoplasm	83801: Endometrioid adenoma, borderline malignancy (D39.1)	Borderline	Ia	NO	NO	YES	YES	19.90	64	12.59	16.03
Type I/BL early	40.95	23.83	Primary borderline epithelial malignant neoplasm	84423: Serous cystadenoma, borderline malignancy (C56)	Borderline	Ic	NO	NO	YES	YES	24.10	55	11.77	22.92
Type I/BL early	39.8	20.31	Primary borderline epithelial malignant neoplasm	84423: Serous cystadenoma, borderline malignancy (C56)	Borderline	Ib	YES	NO	NO	NO	27.81	70	29.50	24.67
Type I/BL early	37.9	23.98	Primary invasive epithelial malignant neoplasm	83803: Endometrioid carcinoma (C56)	Grade 2	IIIc	NO		NO	NO	28.18	56	9.46	25.34
Type I/BL early	33	17.65	Primary invasive epithelial malignant neoplasm	83103: Clear cell adenocarcinoma NOS	High Grade	Ia	NO		NO		37.82	71	13.71	22.73
Type I/BL early	71.83	19.72	Primary borderline epithelial malignant neoplasm	84423: Serous cystadenoma, borderline malignancy (C56)	Borderline	Ic	NO	NO	YES	NO	26.59	73	9.15	21.35
Type I/BL early	51.33	21.38	Primary invasive epithelial malignant neoplasm	81403: Adenocarcinoma NOS (mucinous)	Grade 2	Ia	NO		YES	NO	19.75	69	12.96	20.12
Type I/BL late	8.99	22.43	Primary invasive epithelial malignant neoplasm	83103: Clear cell adenocarcinoma NOS	Grade 3	IIc	NO	NO	NO	NO	23.83	77	12.63	20.87
Type I/BL late	5.14	42.22	Primary borderline epithelial malignant neoplasm	83801: Endometrioid adenoma, borderline malignancy (D39.1)	Borderline	Ia	NO	NO	YES	YES	19.90	70	23.74	28.48
Type I/BL late	3.2	22.21	Primary borderline epithelial malignant neoplasm	84723: Mucinous cystadenoma, borderline malignancy (C56)	Borderline	Ic	NO	NO	NO	NO	23.30	70	17.24	22.58
Type I/BL late	3.24	45.86	Primary invasive epithelial malignant neoplasm	83803: Endometrioid carcinoma (C56)	Grade 2	Ic	NO	NO	YES	NO	30.42	64	34.78	27.37
Type I/BL late	6.59	46.07	Primary borderline epithelial malignant neoplasm	84423: Serous cystadenoma, borderline malignancy (C56)	Borderline	Ia	NO	NO	YES	YES	31.02	58	12.43	23.72
Type I/BL late	8.99	23.41	Primary borderline epithelial malignant neoplasm	84723: Mucinous cystadenoma, borderline malignancy (C56)	Borderline	Ia	NO	NO	YES	YES	22.77	67	22.31	18.22
Type I/BL late	12.14	20.42	Primary invasive epithelial malignant neoplasm	83803: Endometrioid carcinoma (C56)	Grade 2	IIIc	NO		NO	NO	28.18	59	7.37	20.13
Type I/BL late	5.6	21.62	Primary borderline epithelial malignant neoplasm	84423: Serous cystadenoma, borderline malignancy (C56)	Borderline	Ia	NO	NO	NO	YES	29.51	76	25.26	34.27
Type I/BL late	3.24	20.96	Primary borderline epithelial malignant neoplasm	84423: Serous cystadenoma, borderline malignancy (C56)	Borderline	Ic	NO	NO	YES	YES	24.10	59	22.44	17.53
Type I/BL late	11.94	17.53	Primary invasive epithelial malignant neoplasm	83803: Endometrioid carcinoma (C56)	Grade 2	Ia	YES	NO	NO	NO	30.66	74	23.81	35.17
Type I/BL late	2.28	23.1	Primary borderline epithelial malignant neoplasm	84423: Serous cystadenoma, borderline malignancy (C56)	Borderline	Ib	YES	NO	NO	NO	27.81	73	65.34	29.45
Type I/BL late	3.49	41.43	Primary borderline epithelial malignant neoplasm	84623: Papillary serous cystadenoma, borderline malignancy (C56)	Borderline	Ia	NO	NO	NO	NO	23.17	79	15.77	26.62
Type I/BL late	12.46	20.11	Primary invasive epithelial malignant neoplasm	81403: Adenocarcinoma NOS (mucinous)	Grade 2	Ia	NO		YES	NO	19.75	72	14.31	26.89
Type I/BL late	4.48	18.83	Primary borderline epithelial malignant neoplasm	84423: Serous cystadenoma, borderline malignancy (C56)	Borderline	Ic	NO	NO	YES	NO	26.59	79	11.57	21.72
Type I/BL late	6.58	22.03	Primary invasive epithelial malignant neoplasm	83103: Clear cell adenocarcinoma NOS	High Grade	Ia	NO		NO		37.82	73	50.86	36.07
Type I/BL late	4.12	19.71	Primary borderline epithelial malignant neoplasm	83811: Endometrioid adenofibroma, borderline malignancy (D39.1)	Borderline	Ia	NO	NO	NO	NO	32.17	71	34.57	23.91
Type I/BL late	5.53	17.87	Primary invasive epithelial malignant neoplasm	83803: Endometrioid carcinoma (C56)	Grade 2	Ia	NO	NO	YES	NO	26.44	69	44.97	20.62
Type I/BL late	5.93	23.48	Primary invasive epithelial malignant neoplasm	83813: Endometrioid adenofibroma, malignant (C56)	Grade 2	IIa	NO	NO	NO	NO	30.07	63	69.09	29.00
Type I/BL late	12.76	21.11	Primary invasive epithelial malignant neoplasm	83103: Clear cell adenocarcinoma NOS	Grade 3	Ic	YES		YES	NO	28.09	62	11.66	21.23
Type II early	97.46	2.63	Primary invasive epithelial malignant neoplasm	84413: Serous cystadenocarcinoma NOS (C56)	Grade 3	IIa	NO	NO	NO	NO	34.31	59	20.71	29.66
Type II early	64.17	41.22	Primary invasive epithelial malignant neoplasm	84413: Serous cystadenocarcinoma NOS (C56)	High Grade	Ic	YES	YES	YES	YES	29.88	55	11.02	26.22
Type II early	78.57	23.21	Primary invasive epithelial malignant neoplasm	84413: Serous cystadenocarcinoma NOS (C56)	High Grade	III	NO	NO	NO	NO	27.22	73	8.86	18.61
Type II early	62.57	21.96	Primary invasive epithelial malignant neoplasm	84413: Serous cystadenocarcinoma NOS (C56)	Grade 3	Ic	NO	NO	YES	YES	25.01	57	11.32	19.27
Type II early	78.38	23.13	Primary invasive epithelial malignant neoplasm	84413: Serous cystadenocarcinoma NOS (C56)	High Grade	IIIc	YES	NO	NO	NO	20.62	60	24.53	18.48
Type II early	52.68	19.09	Primary invasive epithelial malignant neoplasm	84603: Papillary serous cystadenocarcinoma (C56)	Grade 3	IIc	YES	YES	NO	NO	25.69	65	11.67	29.94
Type II early	65.29	20.05	Primary invasive epithelial malignant neoplasm	83803: Endometrioid carcinoma (C56)	Grade 3	Ic	NO	NO	YES	YES	24.38	61	13.17	20.23
Type II early	37.2	20.28	Primary invasive epithelial malignant neoplasm	84413: Serous cystadenocarcinoma NOS (C56)	High Grade	IIIb	NO	NO	NO	NO	43.23	68	16.92	28.54
Type II early	74.47	3.41	Primary invasive epithelial malignant neoplasm	84413: Serous cystadenocarcinoma NOS (C56)	High Grade	IIa	NO	NO	NO	NO	23.30	59	15.83	32.37
Type II early	58.69	25.99	Primary invasive epithelial malignant neoplasm	89803: Carcinosarcoma NOS	High Grade	IIIc	NO		YES	NO	24.10	60	10.29	14.82
Type II early	66.48	20.68	Primary invasive epithelial malignant neoplasm	84413: Serous cystadenocarcinoma NOS (C56)	High Grade	IIIc	NO	NO	YES	YES	24.87	51	31.21	31.73
Type II early	49.4	46.82	Primary invasive epithelial malignant neoplasm	84413: Serous cystadenocarcinoma NOS (C56)	Grade 3	Ic	NO	NO	NO	NO	29.13	67	16.19	20.79
Type II early	55.28	21.57	Primary invasive epithelial malignant neoplasm	84413: Serous cystadenocarcinoma NOS (C56)	High Grade	IIIc	NO	NO	NO	YES	26.44	63	21.36	27.47
Type II early	90.69	21.77	Primary invasive epithelial malignant neoplasm	84413: Serous cystadenocarcinoma NOS (C56)	Grade 3	Ic	NO	NO	NO	NO	20.80	65	16.74	20.93
Type II early	52.42	24.27	Primary invasive epithelial malignant neoplasm	84413: Serous cystadenocarcinoma NOS (C56)	Grade 3	IIIc	NO	NO	NO	YES	32.79	63	9.17	24.94
Type II early	50.91	26.57	Primary invasive epithelial malignant neoplasm	81403: Adenocarcinoma NOS	Grade 2	II	YES	NO	NO	YES	19.19	66	7.43	25.09
Type II early	36.95	26.13	Primary invasive epithelial malignant neoplasm	84413: Serous cystadenocarcinoma NOS (C56)	Grade 2	Ic	NO	NO	YES	YES	23.83	61	8.53	13.89

Type II early	34.08	19.07	Primary invasive epithelial malignant neoplasm	84413: Serous cystadenocarcinoma NOS (C56)	Grade 3	IIIc	NO		NO	NO	26.09	70	13.39	23.13
Type II early	56.07	25.18	Primary invasive epithelial malignant neoplasm	84413: Serous cystadenocarcinoma NOS (C56)	High Grade	IIIc	NO	NO	YES	YES	31.68	60	13.56	14.71
Type II early	74.33	19.55	Primary invasive epithelial malignant neoplasm	84413: Serous cystadenocarcinoma NOS (C56)	Grade 3	IIIc	YES	NO	NO	YES	24.21	59	19.39	26.99
Type II early	48.96	22.98	Primary invasive epithelial malignant neoplasm	84413: Serous cystadenocarcinoma NOS (C56)	High Grade	IIa	NO	NO	NO	NO	24.21	63	12.56	23.82
Type II early	69.67	4.02	Primary invasive epithelial malignant neoplasm	84413: Serous cystadenocarcinoma NOS (C56)	Grade 3	IIIb	NO	NO	NO	NO	25.20	64	13.10	27.89
Type II early	56.92	22.41	Primary invasive epithelial malignant neoplasm	84413: Serous cystadenocarcinoma NOS (C56)	Grade 3	IIIc	NO		NO	NO	24.31	67	15.07	15.73
Type II early	34.87	21.73	Primary invasive epithelial malignant neoplasm	83803: Endometrioid carcinoma (C56)	Grade 3	IIb	NO	NO	NO	YES	36.05	61	8.94	22.53
Type II early	55.48	26.08	Primary invasive epithelial malignant neoplasm	84603: Papillary serous cystadenocarcinoma (C56)	Grade 3	IIIc	YES	YES	YES	NO	26.89	62	17.35	32.71
Type II early	35.73	26.84	Primary invasive epithelial malignant neoplasm	81403: Adenocarcinoma NOS	Grade 3	Ia	NO	NO	YES	YES	28.14	66	8.56	23.28
Type II early	94.44	20.08	Primary invasive epithelial malignant neoplasm	84413: Serous cystadenocarcinoma NOS (C56)	High Grade	IIc	NO	NO	YES	YES	24.55	55	15.19	25.34
Type II early	73.87	22.44	Primary invasive epithelial malignant neoplasm	83803: Endometrioid carcinoma (C56)	Grade 3	IIIb	NO	NO	NO	YES	23.03	75	11.89	23.02
Type II early	40.69	22.56	Primary invasive epithelial malignant neoplasm	84413: Serous cystadenocarcinoma NOS (C56)	High Grade	IIc	NO	NO	YES	YES	24.89	56	13.05	17.57
Type II early	65.33	25.33	Primary invasive epithelial malignant neoplasm	81403: Adenocarcinoma NOS	High Grade	IIIb	NO	NO	YES	NO	30.42	66	9.57	20.39
Type II late	4.16	24.03	Primary invasive epithelial malignant neoplasm	84413: Serous cystadenocarcinoma NOS (C56)	High Grade	III	NO	NO	NO	NO	27.22	79	102.30	20.95
Type II late	3.2	22.04	Primary invasive epithelial malignant neoplasm	83803: Endometrioid carcinoma (C56)	Grade 3	Ic	NO	NO	YES	YES	24.38	66	30.62	26.07
Type II late	5.9	21.53	Primary invasive epithelial malignant neoplasm	81403: Adenocarcinoma NOS	Grade 2	II	YES	NO	NO	YES	19.19	70	44.59	38.85
Type II late	7.11	22.1	Primary invasive epithelial malignant neoplasm	84413: Serous cystadenocarcinoma NOS (C56)	High Grade	IIIc	NO	NO	YES	YES	31.68	64	34.05	23.88
Type II late	5.34	20.3	Primary invasive epithelial malignant neoplasm	81403: Adenocarcinoma NOS	High Grade	IIIb	NO	NO	YES	NO	30.42	71	56.92	19.61
Type II late	2.97	21.99	Primary invasive epithelial malignant neoplasm	84413: Serous cystadenocarcinoma NOS (C56)	High Grade	IIc	NO	NO	YES	YES	24.55	62	50.51	29.58
Type II late	11.29	22.92	Primary invasive epithelial malignant neoplasm	84413: Serous cystadenocarcinoma NOS (C56)	Grade 3	IIIc	NO		NO	NO	24.31	71	19.56	21.51
Type II late	3.89	23.3	Primary invasive epithelial malignant neoplasm	84413: Serous cystadenocarcinoma NOS (C56)	High Grade	IIc	NO	NO	YES	YES	24.89	59	35.87	23.14
Type II late	3.21	22.85	Primary invasive epithelial malignant neoplasm	83803: Endometrioid carcinoma (C56)	Grade 3	IIb	NO	NO	NO	YES	36.05	64	17.28	32.89
Type II late	3.6	25.6	Primary invasive epithelial malignant neoplasm	84413: Serous cystadenocarcinoma NOS (C56)	High Grade	IIIc	YES	NO	NO	NO	20.62	66	39.99	19.96
Type II late	3.23	40.25	Primary invasive epithelial malignant neoplasm	84413: Serous cystadenocarcinoma NOS (C56)	Grade 3	IIIc	YES	NO	NO	YES	24.21	65	150.30	37.29
Type II late	8.56	18.58	Primary invasive epithelial malignant neoplasm	84413: Serous cystadenocarcinoma NOS (C56)	Grade 3	IIIb	NO	NO	NO	NO	25.20	69	28.82	26.03
Type II late	3.69	22.72	Primary invasive epithelial malignant neoplasm	84603: Papillary serous cystadenocarcinoma (C56)	Grade 3	IIc	YES	YES	NO	NO	25.69	70	99.21	34.22
Type II late	3.24	22.87	Primary invasive epithelial malignant neoplasm	84413: Serous cystadenocarcinoma NOS (C56)	High Grade	IIIb	NO	NO	NO	NO	43.23	71	125.00	21.37
Type II late	5.34	23.79	Primary invasive epithelial malignant neoplasm	84413: Serous cystadenocarcinoma NOS (C56)	High Grade	IIa	NO	NO	NO	NO	24.21	66	31.58	28.76
Type II late	3.93	21.69	Primary invasive epithelial malignant neoplasm	84413: Serous cystadenocarcinoma NOS (C56)	High Grade	IIIc	NO	NO	YES	YES	24.87	56	68.88	27.88
Type II late	11.68	19.56	Primary invasive epithelial malignant neoplasm	84413: Serous cystadenocarcinoma NOS (C56)	Grade 3	Ic	NO	NO	YES	YES	25.01	61	49.89	20.31
Type II late	9.61	21.8	Primary invasive epithelial malignant neoplasm	89803: Carcinosarcoma NOS	High Grade	IIIc	NO		YES	NO	24.10	64	6.94	24.74
Type II late	3.56	19.72	Primary invasive epithelial malignant neoplasm	84413: Serous cystadenocarcinoma NOS (C56)	Grade 3	IIIc	NO		NO	NO	26.09	73	68.31	30.16
Type II late	12.86	22.74	Primary invasive epithelial malignant neoplasm	84413: Serous cystadenocarcinoma NOS (C56)	High Grade	IIa	NO	NO	NO	NO	23.30	64	14.93	25.97
Type II late	14.3	19.13	Primary invasive epithelial malignant neoplasm	83803: Endometrioid carcinoma (C56)	Grade 3	IIIb	NO	NO	NO	YES	23.03	80	10.91	37.33
Type II late	3.43	19.66	Primary invasive epithelial malignant neoplasm	84413: Serous cystadenocarcinoma NOS (C56)	Grade 3	Ic	NO	NO	NO	NO	29.13	71	20.51	18.16
Type II late	6.39	18.74	Primary invasive epithelial malignant neoplasm	84413: Serous cystadenocarcinoma NOS (C56)	Grade 2	Ic	NO	NO	YES	YES	23.83	63	18.43	19.20
Type II late	3.53	45.9	Primary invasive epithelial malignant neoplasm	84413: Serous cystadenocarcinoma NOS (C56)	High Grade	IIIc	NO	NO	NO	YES	26.44	67	180.90	33.97
Type II late	3.7	21.7	Primary invasive epithelial malignant neoplasm	84413: Serous cystadenocarcinoma NOS (C56)	High Grade	Ic	YES	YES	YES	YES	29.88	60	22.43	30.68
Type II late	4.65	21.63	Primary invasive epithelial malignant neoplasm	84413: Serous cystadenocarcinoma NOS (C56)	Grade 3	IIIc	NO	NO	NO	YES	32.79	67	9.19	22.90
Type II late	3.17	21.73	Primary invasive epithelial malignant neoplasm	84413: Serous cystadenocarcinoma NOS (C56)	Grade 3	IIa	NO	NO	NO	NO	34.31	67	74.15	30.45
Type II late	2.87	19.43	Primary invasive epithelial malignant neoplasm	84603: Papillary serous cystadenocarcinoma (C56)	Grade 3	IIIc	YES	YES	YES	NO	26.89	67	548.50	43.64
Type II late	3.5	23.33	Primary invasive epithelial malignant neoplasm	84413: Serous cystadenocarcinoma NOS (C56)	Grade 3	Ic	NO	NO	NO	NO	20.80	73	32.56	20.57
Type II late	3.72	45.38	Primary invasive epithelial malignant neoplasm	81403: Adenocarcinoma NOS	Grade 3	Ia	NO	NO	YES	YES	28.14	69	18.81	18.86
Control early	78.57	26.66					NO	NO	NO	NO	24.80	74	9.07	21.67
Control early	64.17	26.93					NO	NO	YES	NO	23.83	55	11.76	22.85
Control early	69.67	19.54					YES	YES	YES	NO	25.69	64	5.08	22.18
Control early	37.2	17.21					NO	NO	NO	NO	20.83	68	12.26	25.00
Control early	52.68	5.56					YES	YES	YES	YES	30.12	65	7.61	36.87
Control early	97.46	1.72					NO	NO	YES	NO	26.30	60	11.88	29.03
Control early	62.57	41.9					NO	NO	NO	YES	24.96	57	8.59	23.18
Control early	56.92	18.04					NO	NO	YES	NO	23.75	67	11.21	24.58
Control early	36.95	20.3					NO		YES	NO	24.84	60	10.00	32.96
Control early	66.48	21.17					YES	NO	YES	NO	22.54	51	5.08	15.67
Control early	35.73	19.89					NO	NO	NO	YES	20.21	66	9.73	28.91
Control early	52.42	24.25					NO	NO	YES	NO	26.44	63	14.87	19.63
Control early	56.07	25.21					YES	NO	NO	NO	22.69	59	10.62	15.50
Control early	94.44	20.04					YES	YES	YES	YES	22.93	55	11.73	26.49
Control early	34.08	47.94					NO		NO	NO	30.87	70	16.87	29.49
Control early	58.69	21.47					YES	YES	YES	YES	25.60	60	13.74	18.81
Control early	90.69	27.42					NO	NO	NO	YES	23.11	66	13.00	33.45
Control early	55.48	45.3					NO	NO	NO	NO	32.80	62	24.40	19.05
Control early	50.91	24.78					NO	NO	YES	NO	25.51	66	7.65	17.36
Control early	55.28	18.85					NO	NO	YES	YES	21.57	63	18.29	27.01
Control early	40.69	25.06					NO	NO	YES	NO	25.34	55	12.50	22.98

Control early	74.33	24.06	NO	NO	YES	NO	24.80	59	11.43	23.71
Control early	48.96	20.94	YES	NO	YES	YES	24.11	63	15.02	29.74
Control early	78.38	26.54	NO		NO	NO	24.14	60	18.83	19.04
Control early	49.4	18.65	NO	NO	YES	YES	25.03	67	15.39	32.45
Control late	3.72	23.21	NO	NO	NO	YES	20.21	69	9.30	19.50
Control late	3.17	22.6	NO	NO	YES	NO	26.30	66	10.59	25.42
Control late	4.65	23.01	NO	NO	YES	NO	26.44	67	17.38	15.98
Control late	6.39	18.2	NO		YES	NO	24.84	63	14.42	29.09
Control late	5.34	20.44	YES	NO	YES	YES	24.11	66	16.00	37.32
Control late	2.87	19.88	NO	NO	NO	NO	32.80	67	26.51	28.48
Control late	3.23	23.11	NO	NO	YES	NO	24.80	65	13.32	30.56
Control late	9.61	23.06	YES	YES	YES	YES	25.60	64	14.22	28.10
Control late	3.89	23.77	NO	NO	YES	NO	25.34	58	10.82	20.66
Control late	3.43	21.17	NO	NO	YES	YES	25.03	71	14.03	26.86
Control late	3.7	24.09	NO	NO	YES	NO	23.83	60	11.90	23.58
Control late	3.5	22.37	NO	NO	NO	YES	23.11	73	20.39	39.65
Control late	11.68	23.1	NO	NO	NO	YES	24.96	61	12.86	26.84
Control late	3.24	43.68	NO	NO	NO	NO	20.83	71	11.86	20.98
Control late	4.16	22.59	NO	NO	NO	NO	24.80	79	8.57	25.09
Control late	3.53	22.64	NO	NO	YES	YES	21.57	67	19.49	30.24
Control late	11.29	21.93	NO	NO	YES	NO	23.75	71	13.82	21.32
Control late	3.69	19.83	YES	YES	YES	YES	30.12	69	8.26	33.38
Control late	2.97	20.88	YES	YES	YES	YES	22.93	61	11.69	18.31
Control late	3.56	22.2	NO		NO	NO	30.87	73	15.15	23.77
Control late	5.9	26.05	NO	NO	YES	NO	25.51	70	9.08	21.43
Control late	8.56	23.62	YES	YES	YES	NO	25.69	69	8.65	15.49
Control late	3.93	21.67	YES	NO	YES	NO	22.54	56	6.12	17.26
Control late	7.11	20.53	YES	NO	NO	NO	22.69	64	12.20	16.97
Control late	3.6	23.22	NO		NO	NO	24.14	66	14.12	16.71
