# Supplementary material for: Discovery of serum biomarkers of ovarian cancer using complementary proteomic profiling strategies
Source: Proteomics Clin Appl. 2014 Nov 10;8(11-12):982–93. doi: 10.1002/prca.201400063 (PMC4737403; doi:10.1002/prca.201400063)
Supplement: Supplementary file 4 — Table S2 MS‐based protein identifications from 2D‐DIGE profiling of unfactionated, MARS‐depleted and Proteominer‐equalised serum pools. Serum from 131 cases and controls were pooled according to clinical group (M = malignant; B = benign; H = healthy). Pools were left unfractionated (UnF), MARS‐depleted in two separate experiments (MARS1 and 2) or subjected to Proteominer equalisation (PMF) and the samples compared in quadruplicate by 2D‐DIGE using a mixture of all samples as an internal standard. Spots displaying a >1.5‐fold difference in abundance (P <0.05) were targeted for spot picking, tryptic digestion and MS‐based identification using MALDI‐TOF MS (UnF and MARS1) or LC‐MS/MS (MARS2 and PM). Spot number, protein name, IPI accession number, fuctional class, MASCOT score, sequence coverage, number of unique peptides, predicted molecular weight, pI and abundance changes and P values across clinical groups are given. In cases where multiple identifications were made from the same gel spots, all protein groups are reported [file PRCA-8-982-s004.pdf]

Table S2 MS-based protein identifications from 2D-DIGE profiling of unfactionated, MARS-depleted and Proteominer-equalised serum pools. Serum from 131 cases and controls were pooled according to clinical group (M = malignant; B = benign; H = healthy). Pools were left unfractionated (UnF), MARS-depleted in two separate experiments (MARS1 and 2) or subjected to Proteominer equalisation (PMF) and the samples compared in quadruplicate by 2D-DIGE using a mixture of all samples as an internal standard. Spots displaying a >1.5-fold difference in abundance (*P* <0.05) were targeted for spot picking, tryptic digestion and MS-based identification using MALDI-TOF MS (UnF and MARS1) or LC-MS/MS (MARS2 and PM). Spot number, protein name, IPI accession number, fuctional class, MASCOT score, sequence coverage, number of unique peptides, predicted molecular weight, pI and abundance changes and *P* values across clinical groups are given. In cases where multiple identifications were made from the same gel spots, all protein groups are reported.

Experiment	Spot No.	Protein name	IPI No.	Functional class	Score	Seq. Cov (%)	No. peptides	MW	pI	M vs H Av. Ratio	M vs H *P*-value	M vs B Av. Ratio	M vs B *P*-value	B vs H Av. Ratio	B vs H *P*-value
MARS2	73	Alpha-1-antitrypsin (SERPINA1)	IPI00553177	acute phase response	103	40	18	46707	5.37	1.81	0.0055	1.66	0.0068	1.09	0.29
PM	153	Serotransferrin (TF)	IPI00022463	transport	46	4	2	79280	6.81	-1.42	0.0017	-1.65	0.0012	1.16	0.17
PM	160	Serotransferrin (TF)	IPI00022463	transport	59	22	16	79280	6.81	-1.53	0.00017	-1.73	0.00047	1.13	0.25
PM	165	Histidine-rich glycoprotein (HRG)	IPI00022371	coagulation, transport	59	30	16	60510	7.09	-1.31	0.007	-1.60	0.0013	1.22	0.085
PM	174	Serotransferrin (TF)	IPI00022463	transport	59	20	17	79280	6.81	-1.59	6.50E-06	-1.92	5.40E-05	1.21	0.045
PM	208	Pyruvate kinase L (PKLR)	IPI00743713	cellular	63	23	15	64975	7.6	1.09	0.5	1.88	0.0038	-1.72	0.015
PM	215	Serum albumin Isoform 1 (ALB)	IPI00745872	transport, haemostasis	130	10	5	71317	5.92	-1.21	0.02	-1.57	0.00023	1.30	0.013
PM	221	Serum albumin Isoform 1 (ALB)	IPI00745872	transport, haemostasis	813	36	23	71317	5.92	-1.30	0.0043	-1.74	0.00017	1.34	0.0072
PM	222	Serum albumin Isoform 1 (ALB)	IPI00745872	transport, haemostasis	94	34	23	71317	5.92	-1.33	0.0037	-1.56	0.0061	1.17	0.21
PM	224	Serum albumin Isoform 1 (ALB)	IPI00745872	transport, haemostasis	32	167	23	71317	5.92	-1.31	0.0036	-1.54	0.0021	1.17	0.13
PM	226	Serum albumin Isoform 1 (ALB)	IPI00745872	transport, haemostasis	152	42	30	71317	5.92	-1.25	0.00025	-1.58	9.40E-05	1.26	0.0054
PM	228	Serum albumin Isoform 1 (ALB)	IPI00745872	transport, haemostasis	145	44	28	71317	5.92	-1.25	0.012	-1.75	0.00017	1.41	0.00034
PM	231	Serum albumin Isoform 1 (ALB)	IPI00745872	transport, haemostasis	145	46	28	71317	5.92	-1.32	0.011	-1.61	4.60E-05	1.22	0.052
UnF	232	Ceruloplasmin (CP)	IPI00017601	transport	40	2	2	97007	5.31	2.88	0.0095	1.51	0.04	1.34	0.07
UnF	232	Serum albumin (ALB)	IPI00022434	transport, haemostasis	30	8	5	73881	6.33	2.88	0.0095	1.51	0.04	1.34	0.07
UnF	233	Ceruloplasmin (CP)	IPI00017601	transport	38	2	2	122983	5.44	2.59	0.0047	1.40	0.06	1.85	0.02
PM	241	Serum albumin (ALB)	IPI00022434	transport, haemostasis	722	35	20	73881	6.33	-1.31	0.0055	-1.68	0.0002	1.28	0.012
MARS1	242	Ceruloplasmin (CP)	IPI00017601	transport	70	10	7	122983	5.44	-1.05	0.14	1.18	0.21	1.51	0.0086
PM	243	Kinesin-like protein KIF15 (KIF15)	IPI00024975	cellular	68	17	31	161030	5.75	-1.29	0.06	-1.78	0.0048	1.37	0.049
PM	243	Protein FAM117B (FAM117B)	IPI00044665	cellular	63	26	13	29638	11.67	-1.29	0.06	-1.78	0.0048	1.37	0.049
PM	246	Serum albumin Isoform 1 (ALB)	IPI00745872	transport, haemostasis	100	38	23	71317	5.92	-1.19	0.12	-1.62	0.0011	1.37	0.0037
PM	247	Serum albumin Isoform 1 (ALB)	IPI00745872	transport, haemostasis	124	39	26	71317	5.92	-1.27	0.066	-1.93	0.0007	1.52	0.01
PM	248	Serum albumin (ALB)	IPI00022434	transport, haemostasis	1051	45	33	73881	6.33	-1.25	0.12	-1.91	0.0017	1.54	0.014
PM	249	Serum albumin (ALB)	IPI00022434	transport, haemostasis	958	46	29	73881	6.33	-1.25	0.0033	-1.71	1.20E-05	1.37	0.0015
MARS2	252	Serotransferrin (TF)	IPI00022463	transport	81	33	24	77000	6.81	-2.44	1.70E-08	-2.41	9.60E-07	-1.01	0.74
PM	257	Serum albumin (ALB)	IPI00022434	transport, haemostasis	143	33	21	73881	6.33	-1.28	0.033	-1.84	0.0021	1.43	0.028
PM	269	Alpha-1-antitrypsin (SERPINA1)	IPI00553177	acute phase response	96	19	6	46878	5.37	2.82	0.0029	2.20	0.016	1.28	0.44
MARS2	279	Phosphatidylinositol transfer protein alpha isoform (PITPNA)	IPI00216048	cellular	78	25	6	32014	6.11	-2.44	4.20E-07	-2.21	1.10E-06	-1.10	0.026
MARS2	280	Serotransferrin (TF)	IPI00022463	transport	114	38	28	77000	6.81	-1.87	0.0027	-1.82	0.0097	-1.02	0.59
MARS2	281	Immunoglobulin heavy constant mu protein (IGHM)	IPI00477090	immune response	59	8	4	68052	5.89	-2.27	5.60E-07	-2.10	3.50E-07	-1.08	0.032
MARS2	281	Phosphatidylinositol transfer protein alpha isoform (PITPNA)	IPI00216048	cellular	71	11	2	32014	6.11	-2.27	5.60E-07	-2.10	3.50E-07	-1.08	0.032
MARS2	281	Serotransferrin (TF)	IPI00022463	transport	272	20	11	79280	6.81	-2.27	5.60E-07	-2.10	3.50E-07	-1.08	0.032
UnF	284	Serum albumin (ALB)	IPI00022434	transport, haemostasis	29	3	2	73881	6.33	2.86	0.007	1.71	0.008	1.51	0.023
MARS2	289	Antithrombin III (SERPINC1)	IPI00032179	coagulation	47	9	4	53114	6.11	-2.23	3.00E-07	-2.22	5.60E-07	1.00	0.91
MARS2	289	Immunoglobulin heavy constant gamma 1 (IGHG1)	IPI00384938	immune response	124	11	4	53503	6.28	-2.23	3.00E-07	-2.22	5.60E-07	1.00	0.91
PM	289	Immunoglobulin heavy constant gamma 1 (IGHG1)	IPI00384938	immune response	124	11	4	53503	8.74	2.06	2.90E-05	2.64	9.30E-06	-1.28	0.0005
MARS2	289	Phosphatidylinositol transfer protein alpha isoform (PITPNA)	IPI00216048	cellular	82	21	5	32014	6.11	-2.23	3.00E-07	-2.22	5.60E-07	1.00	0.91
PM	291	Alpha-1-antitrypsin (SERPINA1)	IPI00553177	acute phase response	297	30	11	46878	5.37	1.97	0.00038	1.28	0.026	1.54	0.00016
MARS2	297	Serotransferrin (TF)	IPI00022463	transport	315	16	10	79280	6.81	-2.25	4.20E-06	-2.22	5.90E-07	-1.01	0.78
PM	298	Antithrombin III (SERPINC1)	IPI00032179	coagulation	79	37	19	53114	6.11	1.67	0.0007	1.39	0.004	1.20	0.027
PM	299	Antithrombin III (SERPINC1)	IPI00032179	coagulation	59	33	16	53114	6.11	1.84	0.00031	1.19	0.054	1.55	0.00025
PM	300	Alpha-1-antitrypsin (SERPINA1)	IPI00553177	acute phase response	193	20	8	46878	5.37	1.99	0.00027	1.31	0.0016	1.52	0.0062
PM	303	Vitamin D-binding protein precusor (GC/VDBP)	IPI00555812	transport	124	9	6	54526	5.4	1.89	0.00058	1.51	0.01	1.25	0.0033
PM	306	Antithrombin III (SERPINC1)	IPI00032179	coagulation	157	11	7	53114	6.11	1.62	0.0069	1.57	0.021	1.03	0.65
MARS2	323	Serotransferrin (TF)	IPI00022463	transport	146	45	32	77000	6.81	-2.42	3.30E-07	-2.37	8.90E-09	-1.02	0.66
PM	326	Immunoglobulin heavy constant gamma 1 (IGHG1)	IPI00384938	immune response	110	6	3	53503	8.74	-1.71	0.00039	-1.29	0.0034	-1.33	0.017
PM	328	Serum albumin (ALB)	IPI00022434	transport, haemostasis	845	35	20	73881	6.33	-2.65	0.0002	-1.98	0.0008	-1.34	0.0085
PM	329	Immunoglobulin heavy constant gamma 1 (IGHG1)	IPI00384938	immune response	845	35	20	53503	8.74	-1.63	0.0011	-1.32	0.0086	-1.23	0.019
MARS2	332	Serotransferrin (TF)	IPI00022463	transport	140	48	32	77000	6.81	-2.52	6.60E-07	-2.24	2.80E-06	-1.12	0.0072
MARS2	333	Serotransferrin (TF)	IPI00022463	transport	56	26	20	77000	6.81	-2.78	7.50E-08	-2.44	6.50E-08	-1.14	0.00069
MARS2	335	Serum albumin isoform 1 (ALB)	IPI00745872	transport, haemostasis	80	32	22	69321	5.92	-2.17	0.00011	-2.05	0.00018	-1.06	0.2
MARS2	346	Serotransferrin (TF)	IPI00022463	transport	82	31	22	77000	6.81	-2.63	3.20E-07	-2.51	3.30E-07	-1.05	0.28
MARS2	357	Serotransferrin (TF)	IPI00022463	transport	92	8	5	79280	6.81	-1.84	2.80E-06	-1.35	0.00051	-1.36	0.001
MARS2	357	Serum albumin isoform 1 (ALB)	IPI00745872	transport, haemostasis	97	7	5	71317	5.92	-1.84	2.80E-06	-1.35	0.00051	-1.36	0.001

PM	371	Fibrinogen isoform gamma A/B (FGG)	IPI00021891	coagulation	206	22	8	52106	5.37	-6.69	1.90E-05	-5.17	4.40E-05	-1.29	0.0027
PM	381	Complement factor H isoform 2 (CFH)	IPI00218999	complement cascade	430	36	16	52106	6.77	-1.32	0.0002	-1.68	1.80E-05	1.28	0.00092
PM	382	Apolipoprotein AIV (APOA4)	IPI00304273	transport, lipid metabolism	877	50	21	45371	5.28	-1.43	0.00048	-1.83	1.30E-05	1.28	0.0015
UnF	392	Serum albumin (ALB)	IPI00022434	transport, haemostasis	580	39	23	73881	6.33	-1.63	0.015	1.49	0.04	1.24	0.06
PM	393	Apolipoprotein AIV (APOA4)	IPI00304273	transport, lipid metabolism	73	39	15	45344	5.28	1.26	0.012	1.66	0.0003	-1.32	0.012
PM	405	Apolipoprotein AIV (APOA4)	IPI00304273	transport, lipid metabolism	123	45	21	45344	5.28	-1.58	0.0056	-1.70	0.00031	1.08	0.54
PM	405	Kinectin 1 isoform b (KTN1)	IPI00783726	cellular	65	17	32	150545	5.59	-1.58	0.0056	1.08	0.54	-1.70	0.00031
PM	406	Complement factor H-related 1 (CFHR1)	IPI00167093	complement cascade	73	33	14	38766	7.38	-1.25	0.0051	-1.61	7.40E-05	1.29	0.0011
PM	406	Periplakin (PPL)	IPI00298057	cellular	67	18	33	205096	5.44	-1.25	0.0051	-1.61	7.40E-05	1.29	0.0011
MARS1	412	Inter-alpha-trypsin inhibitor heavy chain H4 isoform 1 (ITIH4)	IPI00294193	acute phase response	62	21	20	103489	6.51	2.07	0.018	1.82	0.001	1.29	0.04
PM	431	Complement factor H isoform 1 (CFH)	IPI00029739	complement cascade	159	5	8	143680	6.21	1.62	0.0081	1.32	0.09	1.23	0.044
PM	431	Complement factor H isoform 2 (CFH)	IPI00218999	complement cascade	159	14	8	52711	6.77	1.62	0.0081	1.32	0.09	1.23	0.044
PM	435	Apolipoprotein AIV (APOA4)	IPI00304273	transport, lipid metabolism	158	57	26	45344	5.28	-1.52	0.0017	-1.09	0.24	-1.40	0.00037
PM	435	Pericentrin (PCNT)	IPI00479143	cellular	68	10	49	380644	5.39	-1.52	0.0017	-1.09	0.24	-1.40	0.00037
PM	444	Ral GAP subunit alpha-1 isoform 3 (RALGAPA1)	IPI00646904	cellular	68	23	15	125169	5.82	1.24	0.01	1.97	9.50E-05	-1.59	0.00066
UnF	458	Immunoglobulin heavy constant mu protein (IGHM)	IPI00385264	immune response	54	8	3	43543	5.13	2.01	0.043	1.18	0.77	1.62	0.26
PM	458	Serum albumin isoform 2 (ALB)	IPI00384697	transport, haemostasis	537	25	14	48641	5.97	-1.29	0.00095	-1.75	1.10E-05	1.35	0.00086
MARS1	459	Alpha-1B-glycoprotein (A1BG)	IPI00022895	unknown	52	7	2	54809	5.58	2.51	0.023	2.18	0.32	1.15	0.67
MARS1	459	Histidine-rich glycoprotein (HRG)	IPI00022371	coagulation, transport	137	10	5	60510	7.09	2.51	0.023	2.18	0.32	1.15	0.67
MARS1	459	Inter-alpha (Globulin) inhibitor H4 variant	IPI00556036	acute phase response	401	22	16	76971	5.72	2.51	0.023	2.18	0.32	1.15	0.67
MARS1	459	Prothrombin (F2)	IPI00019568	coagulation	95	7	4	71475	5.64	2.51	0.023	2.18	0.32	1.15	0.67
MARS1	459	Vitamin K-dependent protein S (PROS1)	IPI00294004	transport	46	4	3	77127	5.48	2.51	0.023	2.18	0.32	1.15	0.67
PM	461	Adenomatosis polyposis coli 2 (APC2)	IPI00025190	cellular	64	11	31	245966	9.08	-1.26	0.0015	-1.53	8.90E-05	1.21	0.0035
PM	461	Apolipoprotein E (APOE)	IPI00021842	transport, lipid metabolism	68	41	16	36246	5.65	-1.26	0.0015	-1.53	8.90E-05	1.21	0.0035
MARS2	461	Serum albumin isoform 1 (ALB)	IPI00745872	transport, haemostasis	96	32	22	71317	5.92	1.77	1.50E-06	-2.22	1.30E-06	3.94	1.40E-08
MARS1	464	Afamin (AFM)	IPI00019943	transport	92	8	3	70963	5.64	2.65	0.015	1.91	0.16	1.30	0.44
MARS1	464	Alpha-1B-glycoprotein (A1BG)	IPI00022895	unknown	189	17	6	54809	5.58	2.65	0.015	1.91	0.16	1.30	0.44
MARS1	464	Histidine-rich glycoprotein (HRG)	IPI00022371	coagulation, transport	242	19	9	60510	7.09	2.65	0.015	1.91	0.16	1.30	0.44
MARS1	464	Inter-alpha-trypsin inhibitor heavy chain H4 isoform 1 (ITIH4)	IPI00294193	acute phase response	289	11	12	103489	6.51	2.65	0.015	1.91	0.16	1.30	0.44
MARS1	464	Prothrombin (F2)	IPI00019568	coagulation	86	7	4	71475	5.64	2.65	0.015	1.91	0.16	1.30	0.44
MARS2	464	Serum albumin (ALB)	IPI00022434	transport, haemostasis	67	26	19	73881	6.33	1.04	0.44	-2.02	2.10E-05	2.09	6.30E-05
MARS1	464	Vitamin K-dependent protein S (PROS1)	IPI00294004	transport	61	4	3	77127	5.48	2.65	0.015	1.91	0.16	1.30	0.44
MARS2	465	Serum albumin (ALB)	IPI00022434	transport, haemostasis	108	36	32	62166	6.33	1.40	0.0057	-2.20	5.50E-05	3.07	4.20E-05
MARS1	468	Alpha-2-macroglobulin (A2M)	IPI00478003	coagulation	102	5	6	164600	6	-2.43	0.0056	-2.08	0.0074	-1.09	0.22
MARS1	468	Complement factor B isoform 1 (CFB Fragment)	IPI00019591	complement cascade	123	5	4	86847	6.67	-2.43	0.0056	-2.08	0.0074	-1.09	0.22
MARS1	471	Alpha-1B-glycoprotein (A1BG)	IPI00022895	unknown	86	10	4	54809	5.58	1.89	0.044	1.11	0.64	-1.21	0.33
MARS1	471	Complement component C9 precursor	IPI00022395	complement cascade	196	16	9	64615	5.43	1.89	0.044	1.11	0.64	-1.21	0.33
MARS1	471	Histidine-rich glycoprotein (HRG)	IPI00022371	coagulation, transport	364	21	11	60510	7.09	1.89	0.044	1.11	0.64	-1.21	0.33
MARS1	471	Inter-alpha (Globulin) inhibitor H4 variant	IPI00556036	acute phase response	261	13	10	76971	5.72	1.89	0.044	1.11	0.64	-1.21	0.33
MARS1	472	Inter-alpha-trypsin inhibitor heavy chain H4 isoform 2 (ITIH4)	IPI00218192	acute phase response	64	22	19	101488	6.21	-3.83	0.029	-1.09	0.72	1.78	0.36
PM	475	Apolipoprotein E (APOE)	IPI00021842	transport, lipid metabolism	373	32	12	36246	5.65	1.53	0.00047	1.35	0.011	1.13	0.063
MARS2	477	Serum albumin isoform 1 (ALB)	IPI00745872	transport, haemostasis	78	32	24	61945	5.92	1.78	1.90E-06	-2.26	5.60E-06	4.02	1.10E-07
PM	478	Apolipoprotein E (APOE)	IPI00021842	transport, lipid metabolism	91	52	22	36841	5.65	1.77	0.0059	1.33	0.047	1.33	0.065
PM	496	Apolipoprotein E (APOE)	IPI00021842	transport, lipid metabolism	685	48	15	36246	5.65	1.05	0.37	1.57	0.00027	-1.49	7.80E-05
PM	499	Apolipoprotein E (APOE)	IPI00021842	transport, lipid metabolism	773	46	14	36246	5.65	1.20	0.021	1.51	0.0012	-1.26	0.017
PM	501	Complement C4A (C4A)	IPI00032258	complement cascade	168	5	9	194247	6.65	2.23	5.50E-05	1.92	0.00016	1.16	0.048
PM	507	Complement factor H-related protein 2 (CFHR2)	IPI00006154	complement cascade	206	17	4	28734	6.52	5.38	0.0019	5.17	0.00053	1.04	0.75
PM	511	Serotransferrin (TF)	IPI00022463	transport	325	26	16	79280	6.81	1.26	0.002	2.11	1.90E-05	-1.67	0.0006
PM	517	Immunoglobulin lambda protein (IGL)	IPI00658130	immune response	106	13	4	25347	8.14	1.96	7.40E-05	1.98	2.70E-05	-1.01	0.91
MARS2	523	Alpha-1-antichymotrypsin isoform 1 (SERPINA3)	IPI00550991	acute phase response	92	34	14	60850	5.42	2.00	2.90E-07	1.73	2.20E-06	1.16	0.0081
PM	562	Apolipoprotein AI (APOA1)	IPI00021841	transport, lipid metabolism	147	49	15	30759	5.56	-1.53	0.0072	-1.17	0.23	-1.31	0.094
MARS1	566	Alpha-1B-glycoprotein (A1BG)	IPI00022895	unknown	72	37	16	54809	5.58	1.38	0.068	1.50	0.032	1.08	0.43
MARS1	566	Complement component C9 precursor	IPI00022395	complement cascade	63	24	18	64615	5.43	1.38	0.068	1.52	0.032	1.08	0.43
PM	571	Apolipoprotein AI (APOA1)	IPI00021841	transport, lipid metabolism	236	85	32	30759	5.56	-1.90	0.00068	-1.40	0.029	-1.36	0.05
PM	577	Utrophin fragment (UTRN)	IPI00009329	cellular	70	11	49	396472	5.21	1.39	0.00045	-1.11	0.034	1.54	8.70E-05
MARS2	585	Immunoglobulin heavy chain constant region alpha 1 (IGHA1)	IPI00386879	immune response	97	6	3	54195	6.46	1.01	0.98	-1.57	0.018	1.59	0.01
UnF	611	Alpha-1-antichymotrypsin isoform 3 (SERPINA3)	IPI00847635	acute phase response	65	12	6	47792	5.33	1.55	0.0092	1.93	0.03	1.17	0.28
UnF	611	Kininogen 1 isoform LMW (KNG1)	IPI00215894	coagulation, acute phase response	37	4	2	48936	6.29	1.55	0.0092	1.93	0.03	1.17	0.28
UnF	614	Serum albumin (ALB)	IPI00022434	transport, haemostasis	251	21	13	71317	5.92	-1.60	0.028	1.02	0.45	1.03	0.41
UnF	618	Alpha-1-antichymotrypsin isoform 1 (SERPINA3)	IPI00550991	acute phase response	65	4	2	50737	5.42	2.95	0.0093	1.31	0.34	1.50	0.21
UnF	636	Immunoglobulin heavy chain constant region alpha 1 (IGHA1)	IPI00719233	immune response	106	7	4	54150	6.78	2.04	0.026	1.31	0.06	2.53	0.004
UnF	636	Serum albumin (ALB)	IPI00022434	transport, haemostasis	229	15	11	71317	5.92	2.04	0.026	1.31	0.06	2.53	0.004
MARS2	644	Alpha-1-antitrypsin (SERPINA1)	IPI00790784	acute phase response	72	29	14	40238	4.98	2.04	7.50E-08	1.72	4.80E-06	1.18	0.0011
MARS2	644	Phenylalanyl-tRNA synthetase beta chain (FARSB)	IPI00300074	cellular	63	22	18	66715	6.4	2.04	7.50E-08	1.72	4.80E-06	1.18	0.0011
UnF	648	Keratin, type 1 cytoskeletal 9	IPI00019359	cellular	68	5	2	62131	5.14	1.80	0.17	1.13	0.67	1.89	0.04
UnF	648	Serum albumin (ALB)	IPI00022434	transport, haemostasis	322	24	18	73881	6.33	1.80	0.17	1.13	0.67	1.89	0.04

UnF	654	Immunoglobulin heavy chain constant region alpha 1 (IGHA1)	IPI00719233	immune response	65	8	5	54150	6.78	1.99	0.036	1.22	0.08	2.20	0.015
UnF	654	Serum albumin (ALB)	IPI00022434	transport, haemostasis	51	5	7	73881	6.33	1.99	0.036	1.22	0.08	2.20	0.015
MARS1	657	Complement factor B isoform 1 (CFB Fragment)	IPI00019591	complement cascade	116	3	7	86847	6.67	-1.43	0.087	1.90	0.015	1.00	0.99
MARS2	664	Alpha-1-antitrypsin (SERPINA1)	IPI00553177	acute phase response	136	43	24	55397	5.37	1.84	2.60E-07	1.74	2.60E-06	1.06	0.041
MARS2	664	Protein Daple isoform 1 (CCDC88C)	IPI00740019	cellular	82	18	37	229215	5.87	1.84	2.60E-07	1.74	2.60E-06	1.06	0.041
MARS2	664	Ras-related protein Rab-2B (RAB2B)	IPI00102896	cellular	66	57	11	24427	7.68	1.84	2.60E-07	1.74	2.60E-06	1.06	0.041
MARS2	664	Vinculin isoform 2 (VCL)	IPI00307162	cellular	71	23	26	124292	5.5	1.84	2.60E-07	1.74	2.60E-06	1.06	0.041
UnF	678	Immunoglobulin heavy constant mu protein (IGHM)	IPI00472610	immune response	73	4	2	52665	7.5	2.40	0.0065	-1.04	0.82	2.12	0.15
MARS2	680	Alpha-1-antitrypsin (SERPINA1)	IPI00553177	acute phase response	69	32	16	46707	5.37	1.68	1.60E-07	1.53	2.40E-05	1.09	0.04
UnF	691	Immunoglobulin heavy constant gamma 3 (IGHG3 Fragment)	IPI00829940	immune response	128	11	4	38769	8.36	4.45	0.0011	2.57	0.005	3.62	0.004
UnF	691	Immunoglobulin heavy constant mu protein (IGHM)	IPI00472610	immune response	140	13	4	52665	7.5	4.45	0.0011	2.57	0.005	3.62	0.004
UnF	693	Immunoglobulin heavy constant gamma 1 (IGHG1)	IPI00423464	immune response	220	16	8	53011	8.84	4.69	0.0096	2.23	0.02	3.12	0.025
UnF	693	Immunoglobulin heavy constant mu protein (IGHM)	IPI00472610	immune response	105	8	3	52665	7.5	4.69	0.0096	2.23	0.02	3.12	0.025
MARS2	712	Alpha-1-antitrypsin (SERPINA1)	IPI00553177	acute phase response	145	49	23	46707	5.37	1.62	9.90E-07	1.52	6.30E-05	1.06	0.17
MARS2	712	T-box transcription factor isoform 1 (TBX3)	IPI00298944	cellular	76	28	18	77529	8.48	1.62	9.90E-07	1.52	6.30E-05	1.06	0.17
MARS2	712	Vinculin isoform 2 (VCL)	IPI00307162	cellular	70	19	25	124292	5.5	1.62	9.90E-07	1.52	6.30E-05	1.06	0.17
UnF	734	Alpha-2-HS-glycoprotein (AHSG)	IPI00022431	acute phase response, mineral balance	136	16	6	40098	5.43	1.65	0.01	1.00	0.71	1.36	0.04
UnF	740	Immunoglobulin heavy constant gamma 2 (IGHG2 Fragment)	IPI00399007	immune response	32	5	2	46716	7.63	3.24	0.012	1.28	0.5	3.25	0.051
UnF	750	Coagulation factor VII	IPI00329555	coagulation	120	5	8	77386	6.6	3.78	0.0033	1.42	0.33	3.13	0.042
UnF	750	Immunoglobulin heavy constant gamma 2 (IGHG2 Fragment)	IPI00399007	immune response	166	12	5	46716	7.63	3.78	0.0033	1.42	0.33	3.13	0.042
UnF	750	Immunoglobulin heavy constant mu protein (IGHM)	IPI00472610	immune response	107	7	3	52665	7.5	3.78	0.0033	1.42	0.33	3.13	0.042
UnF	753	Coagulation factor VII	IPI00329555	coagulation	67	8	6	77386	6.6	3.87	0.007	1.92	0.015	3.94	0.009
UnF	753	Immunoglobulin heavy constant mu protein (IGHM)	IPI00472610	immune response	101	9	4	52665	7.5	3.87	0.007	1.92	0.015	3.94	0.009
UnF	763	Immunoglobulin heavy constant gamma 1 (IGHG1)	IPI00384938	immune response	97	7	3	53503	8.74	4.91	0.014	2.02	0.24	2.14	0.17
UnF	763	Immunoglobulin heavy constant mu protein (IGHM)	IPI00472610	immune response	245	9	5	52665	7.5	4.91	0.014	2.02	0.24	2.14	0.17
UnF	765	Immunoglobulin heavy constant gamma 1 (IGHG1)	IPI00423464	immune response	425	29	13	53011	8.84	4.32	0.0081	1.85	0.19	2.67	0.076
UnF	765	Immunoglobulin heavy constant mu protein (IGHM)	IPI00472610	immune response	315	22	7	52665	7.5	4.32	0.0081	1.85	0.19	2.67	0.076
MARS1	766	Alpha-2-macroglobulin (A2M)	IPI00478003	coagulation	62	13	19	164600	6	-1.73	0.037	1.13	0.48	1.00	0.99
UnF	782	Apolipoprotein AIV (APOA4)	IPI00304273	transport, lipid metabolism	54	10	4	45371	5.28	1.24	0.095	1.57	0.027	1.14	0.3
UnF	782	Haptoglobin (HP)	IPI00431645	acute phase response	98	21	7	31647	8.84	1.24	0.095	1.57	0.027	1.14	0.3
UnF	782	Serum albumin (ALB)	IPI00022434	transport, haemostasis	36	4	3	73881	6.33	1.24	0.095	1.57	0.027	1.14	0.3
UnF	800	Haptoglobin (HP)	IPI00431645	acute phase response	76	12	5	31647	8.84	2.92	0.053	2.58	0.022	1.29	0.47
UnF	808	Haptoglobin (HP)	IPI00431645	acute phase response	363	41	16	31647	8.84	3.37	0.012	2.69	0.021	1.25	0.35
UnF	814	Alpha-1-antitrypsin (SERPINA1)	IPI00553177	acute phase response	159	21	6	46878	5.37	2.94	0.01	2.43	0.009	1.27	0.06
UnF	814	Haptoglobin (HP)	IPI00431645	acute phase response	434	41	16	31647	8.84	2.94	0.01	2.43	0.009	1.27	0.06
UnF	822	Haptoglobin (HP)	IPI00431645	acute phase response	162	25	10	31647	8.48	2.71	0.032	2.35	0.0086	1.38	0.24
UnF	826	Apolipoprotein AIV (APOA4)	IPI00304273	transport, lipid metabolism	37	10	4	45371	5.28	2.29	0.072	2.21	0.018	1.38	0.32
UnF	826	Haptoglobin (HP)	IPI00431645	acute phase response	453	43	19	31647	8.48	2.29	0.072	2.21	0.018	1.38	0.32
UnF	826	Serum albumin (ALB)	IPI00022434	transport, haemostasis	93	7	5	73881	6.33	2.29	0.072	2.21	0.018	1.38	0.32
UnF	833	Haptoglobin (HP)	IPI00431645	acute phase response	555	43	21	31647	8.48	1.55	0.014	-1.08	0.72	-1.21	0.57
UnF	842	Complement C3 precursor	IPI00739237	complement cascade	53	7	3	45642	4.94	2.03	0.011	1.34	0.11	1.81	0.018
UnF	848	Haptoglobin (HP)	IPI00431645	acute phase response	54	8	5	31647	8.48	2.46	0.014	1.93	0.36	-1.02	0.87
UnF	850	Haptoglobin (HP)	IPI00431645	acute phase response	115	21	8	31647	8.48	2.20	0.024	2.31	0.036	1.29	0.26
MARS2	930	Haptoglobin (HP)	IPI00431652	acute phase response	70	45	16	31362	8.48	2.09	0.00026	2.03	0.00024	1.03	0.67
MARS2	948	Apolipoprotein AIV (APOA4)	IPI00304273	transport, lipid metabolism	83	9	4	45371	5.28	1.77	1.80E-05	1.48	6.40E-05	1.20	0.0031
MARS2	971	Apolipoprotein AIV (APOA4)	IPI00304273	transport, lipid metabolism	190	55	24	44044	5.24	3.25	1.10E-08	1.79	4.20E-06	1.82	6.60E-06
MARS2	973	Haptoglobin (HP)	IPI00431648	acute phase response	497	39	17	31647	8.48	1.99	5.20E-06	1.63	4.20E-05	1.22	0.0041
MARS2	974	Alpha-1-acid glycoprotein 2 (ORM2)	IPI00022895	transport	52	7	2	54309	5.58	-1.44	0.00017	1.10	0.063	-1.59	0.00019
MARS2	991	Haptoglobin (HP)	IPI00431651	acute phase response	63	43	14	31647	8.48	2.81	5.30E-08	1.85	2.00E-06	1.52	8.70E-06
MARS2	1010	Haptoglobin (HP)	IPI00431649	acute phase response	64	38	14	31362	8.48	2.58	1.10E-07	1.71	8.10E-06	1.51	2.60E-05
MARS2	1042	Haptoglobin (HP)	IPI00431650	acute phase response	82	42	17	38941	6.13	2.69	2.80E-07	1.78	5.00E-05	1.51	0.00024
MARS2	1044	Haptoglobin (HP)	IPI00431647	acute phase response	61	39	14	31362	8.48	2.03	1.90E-05	1.37	0.0019	1.48	0.0005
MARS2	1047	Haptoglobin (HP)	IPI00431646	acute phase response	64	39	14	38427	6.13	1.74	3.60E-06	1.22	0.004	1.43	0.00045
MARS2	1074	Serum albumin (ALB)	IPI00022434	transport, heamostasis	30	3	2	39529	5.38	2.54	2.90E-05	1.76	4.80E-06	1.44	0.0042
MARS2	1137	Alpha-1-antitrypsin (SERPINA1)	IPI00553177	acute phase response	631	38	21	46878	5.37	1.61	9.30E-05	-1.04	0.2	1.68	0.0002
MARS2	1193	Alpha-1-antitrypsin (SERPINA1)	IPI00553177	acute phase response	40	6	2	46878	5.37	1.55	4.50E-05	1.21	0.014	1.28	0.0072
MARS2	1280	Immunoglobulin kappa variable 1-5 (IGKV1)	IPI00430820	immune response	57	18	3	26034	5.74	-1.22	0.0014	-1.71	1.40E-06	1.41	1.20E-05
MARS2	1425	Haptoglobin-related protein isoform 1 (HPR)	IPI00477597	unknown	178	8	6	39496	6.42	-1.79	0.003	-1.69	0.0047	-1.05	0.3

Not identified

Expt	Spot No.	Protein name	IPI No.	Functional class	Score	Seq. Cov (%)	No. peptides	MW	pI	M vs H Av. Ratio	M vs H *P*-value	M vs B Av. Ratio	M vs B *P*-value	B vs H Av. Ratio	B vs H *P*-value
UnF	282	Not identified								2.62	0.002	1.69	0.035	1.10	0.4
UnF	283	Not identified								2.40	0.0095	1.58	0.081	1.09	0.59

UnF	303	Not identified	2.04	0.044	1.46	0.32	1.07	0.72
UnF	307	Not identified	2.08	0.0062	1.99	0.007	1.40	0.02
UnF	361	Not identified	1.63	0.0054	1.79	0.004	1.12	0.13
UnF	462	Not identified	1.92	0.011	-1.07	0.76	1.76	0.21
UnF	499	Not identified	1.39	0.25	2.72	0.031	2.65	0.015
UnF	646	Not identified	4.09	0.0085	1.90	0.012	2.12	0.015
UnF	710	Not identified	2.05	0.041	1.29	0.37	1.41	0.18
UnF	749	Not identified	1.58	0.042	1.14	0.345	1.90	0.029
UnF	759	Not identified	4.34	0.0078	1.80	0.18	2.85	0.059
UnF	766	Not identified	4.69	0.0091	2.02	0.2	2.17	0.14
UnF	852	Not identified	1.55	0.054	2.82	0.025	-1.10	0.65
UnF	855	Not identified	1.75	0.028	3.15	0.002	-1.12	0.23
UnF	898	Not identified	3.50	0.024	1.81	0.051	4.85	0.001
UnF	899	Not identified	3.43	0.015	1.68	0.06	4.62	0.00024
UnF	928	Not identified	3.48	0.022	1.97	0.01	4.95	0.003
MARS2	76	Not Identified	1.65	0.0013	1.55	0.0031	1.06	0.37
MARS2	220	Not Identified	-1.44	0.01	1.15	0.2	-1.66	0.00033
MARS2	250	Not Identified	-1.96	7.00E-07	-2.00	2.10E-07	1.02	0.62
MARS2	296	Not Identified	-2.00	1.20E-05	-1.98	6.80E-06	-1.01	0.79
MARS2	352	Not Identified	-2.14	0.00079	-1.68	0.0053	-1.27	0.0076
MARS2	407	Not Identified	-1.77	0.00055	-1.65	0.00084	-1.07	0.06
MARS2	452	Not Identified	-1.12	0.24	-2.14	2.30E-05	1.91	0.00046
MARS2	457	Not Identified	1.70	2.30E-05	-1.96	4.70E-07	3.34	2.00E-07
MARS2	459	Not Identified	1.42	0.0003	-2.01	3.60E-06	2.85	9.40E-07
MARS2	537	Not Identified	1.56	7.50E-08	1.56	2.30E-09	-1.00	0.99
MARS2	541	Not Identified	-1.76	0.0022	-2.41	0.00014	1.37	0.0084
MARS2	609	Not Identified	-1.68	0.0065	-2.06	0.0012	1.23	0.00067
MARS2	769	Not Identified	-1.74	6.30E-06	-1.25	0.00015	-1.39	0.00015
MARS2	774	Not Identified	2.05	0.0015	1.23	0.012	1.66	0.0065
MARS2	885	Not Identified	2.28	2.70E-06	2.13	1.20E-05	1.07	0.14
MARS2	888	Not Identified	-1.41	0.048	-2.03	0.009	1.44	0.0016
MARS2	926	Not Identified	3.04	1.60E-05	1.99	1.40E-07	1.53	0.0001
MARS2	950	Not Identified	2.48	1.60E-07	1.64	6.80E-06	1.51	6.10E-05
MARS2	1021	Not Identified	2.25	3.50E-07	1.53	2.20E-05	1.47	6.40E-05
MARS2	1093	Not Identified	2.65	3.10E-08	1.71	9.90E-06	1.55	5.20E-05
MARS2	1117	Not Identified	2.08	4.60E-07	1.39	0.00027	1.50	2.30E-05
MARS2	1158	Not Identified	-1.40	0.0012	-1.83	5.50E-06	1.31	0.0014
MARS2	1160	Not Identified	1.40	7.30E-06	-1.10	0.011	1.55	3.20E-07
MARS2	1186	Not Identified	-1.57	0.00029	-1.29	0.0057	-1.22	0.0023
MARS2	1249	Not Identified	-1.52	0.0033	-1.77	9.80E-05	1.16	0.11
MARS2	1253	Not Identified	-1.60	0.0076	-1.21	0.15	-1.32	0.007
MARS2	1265	Not Identified	-1.61	0.00037	-2.05	2.00E-06	1.27	0.0079
MARS2	1313	Not Identified	-1.23	0.4	-6.89	0.00026	5.61	2.70E-05
MARS2	1325	Not Identified	-1.27	0.011	-1.77	5.30E-05	1.39	0.0017
MARS2	1351	Not Identified	-1.35	0.00023	-1.61	0.00027	1.19	0.04
MARS2	1360	Not Identified	-1.44	0.00041	-1.69	0.00011	1.17	0.021
MARS2	1394	Not Identified	-2.07	0.0023	-1.77	0.0055	-1.17	0.18
MARS2	1402	Not Identified	1.52	0.0012	1.06	0.37	1.43	0.0003
MARS2	1417	Not Identified	-2.59	7.20E-06	-2.69	1.90E-05	1.04	0.53
MARS2	1439	Not Identified	2.06	1.00E-06	1.60	2.30E-06	1.29	0.00071
MARS2	1441	Not Identified	2.15	7.50E-06	1.14	0.099	1.88	1.20E-05
MARS2	1442	Not Identified	2.53	2.10E-08	1.46	4.40E-06	1.73	3.00E-06
MARS2	1445	Not Identified	2.39	9.50E-09	1.56	1.10E-06	1.53	5.10E-06
PM	149	Not Identified	-1.24	0.051	-1.59	0.0019	1.28	0.018
PM	157	Not Identified	-1.31	0.00016	-1.63	0.00018	1.25	0.014
PM	164	Not Identified	-1.48	0.00021	-1.90	2.50E-05	1.28	0.022
PM	192	Not Identified	1.01	0.92	1.97	0.0083	-1.96	0.0014
PM	217	Not Identified	-1.26	0.014	-1.57	0.0005	1.24	0.048
PM	285	Not Identified	2.19	0.00028	1.49	0.0012	1.47	0.0072
PM	319	Not Identified	-2.35	0.0014	-1.61	0.012	-1.46	0.0022
PM	339	Not Identified	1.35	0.02	1.57	0.0019	-1.16	0.13
PM	360	Not Identified	-2.93	9.00E-07	-1.84	2.70E-05	-1.59	0.00044
PM	445	Not Identified	1.20	0.019	1.76	0.00042	-1.46	0.012
PM	500	Not Identified	-1.23	0.014	-1.66	0.00029	1.35	0.00065
PM	506	Not Identified	-1.24	0.024	-1.72	0.0012	1.39	0.0089
